# Epitaxial growth of inch-scale single-crystal transition metal dichalcogenides through the patching of unidirectionally orientated ribbons

**DOI:** 10.1038/s41467-022-30900-9

**Published:** 2022-06-10

**Authors:** Pengfei Yang, Dashuai Wang, Xiaoxu Zhao, Wenzhi Quan, Qi Jiang, Xuan Li, Bin Tang, Jingyi Hu, Lijie Zhu, Shuangyuan Pan, Yuping Shi, Yahuan Huan, Fangfang Cui, Shan Qiao, Qing Chen, Zheng Liu, Xiaolong Zou, Yanfeng Zhang

**Affiliations:** 1 School of Materials Science and Engineering, Peking University, Beijing, 100871 People’s Republic of China; 2 Shenzhen Geim Graphene Center and Tsinghua-Berkeley Shenzhen Institute (TBSI), Tsinghua University, Shenzhen, 518055 People’s Republic of China; 3 School of Materials Science and Engineering, Nanyang Technological University, Singapore, 639798 Singapore; 4 State Key Laboratory of Functional Materials for Informatics, Shanghai Institute of Microsystem and Information Technology, Chinese Academy of Sciences, Shanghai, 200050 People’s Republic of China; 5 Key Laboratory for the Physics and Chemistry of Nanodevices, Department of Electronics, Peking University, Beijing, 100871 People’s Republic of China; 6 Academy for Advanced Interdisciplinary Studies, Peking University, Beijing, 100871 People’s Republic of China

**Keywords:** Two-dimensional materials, Synthesis and processing

## Abstract

Two-dimensional (2D) semiconductors, especially transition metal dichalcogenides (TMDs), have been envisioned as promising candidates in extending Moore’s law. To achieve this, the controllable growth of wafer-scale TMDs single crystals or periodic single-crystal patterns are fundamental issues. Herein, we present a universal route for synthesizing arrays of unidirectionally orientated monolayer TMDs ribbons (e.g., MoS_2_, WS_2_, MoSe_2_, WSe_2_, MoS_x_Se_2-x_), by using the step edges of high-miller-index Au facets as templates. Density functional theory calculations regarding the growth kinetics of specific edges have been performed to reveal the morphological transition from triangular domains to patterned ribbons. More intriguingly, we find that, the uniformly aligned TMDs ribbons can merge into single-crystal films through a one-dimensional edge epitaxial growth mode. This work hereby puts forward an alternative pathway for the direct synthesis of inch-scale uniform monolayer TMDs single-crystals or patterned ribbons, which should promote their applications as channel materials in high-performance electronics or other fields.

## Abstract (web summary)

Here, the authors report the direct growth of periodic arrays of 2D semiconductor ribbons by exploiting the step edges of high-miller-index Au facets, showing potential for 2D electronic devices. The synthesized ribbons could also be merged to obtain wafer-scale single-crystal monolayers.

## Introduction

Two-dimensional (2D) semiconducting transition metal dichalcogenides (TMDs) have attracted tremendous interest for their broad-range applications in electronics, optoelectronics, catalysis, etc.^1–3^. The morphology or dimensionality of TMDs is a critical factor to determine their physical properties. For instance, one-dimension (1D) monolayer MoS_2_ ribbon is predicted to possess novel properties such as metallic edge states^4^, 1D confined plasmons^5^, ferromagnetic behaviors^6^, etc. Besides, it can present improved catalytic property in hydrogen evolution reaction (HER) due to the abundant active edge sites^7^, and more importantly, maintained high carrier mobility^8^. So far, the fabrication of monolayer TMDs ribbons has largely depended on micro-nanofabrication approaches, by using electron beam or optical lithography patterning techniques^9^. However, these top-down methods are evidently tedious and usually need to start with large-area uniform continuous films or crystals.

Recently, several strategies have been developed for the direct synthesis of monolayer TMDs ribbons^10^, such as Na-Mo-O droplets driven growth on NaCl single crystals^11^, substrate-directed synthesis on phosphine pre-treated Si(001) substrates^12^, as well as ledge-directed epitaxy on β-Ga_2_O_3_ (100)^13^. These strategies have individually achieved the control of their thickness, orientation and dimensionality, nevertheless, the synthesis of TMDs ribbons possessing all the above advantages has not been realized.

Wafer-scale monolayer TMDs single crystals, characterized by intrinsically high crystallinity and extremely uniform property, have long been pursued. However, due to the non-centrosymmetric structures of TMDs, antiparallel domains and twin boundaries usually evolved on most growth substrates^14–16^, similar to those encountered in the preparation of *h*-BN. Lately, wafer-scale growth of monolayer *h*-BN single crystal was achieved by using liquid Au as substrate via the self-collimation of circular *h*-BN grains^17^. Wafer-scale monolayer *h*-BN single crystals were also realized on Cu(110)^18^ and Cu(111)^19^ substrates, where the nucleation and growth of *h*-BN domains were dominantly guided by the substrate-step edges. Very recently, our group realized the epitaxial growth of inch-scale monolayer MoS_2_ single crystals on Au(111) films^20^, demonstrating the potential for the unidirectionally oriented growth of TMDs grains. Despite these achievements, the successful attempts regarding the growth of wafer-scale monolayer TMDs single crystals on Au substrates remain very limited^21,22^. Meanwhile, the step-edge-guided growth of single-crystal TMDs has also been explored on insulating sapphire substrates^23–26^. Particularly, inch-scale single-crystal MoS_2_ and WS_2_ monolayers have been achieved on C-A and A plane sapphire substrates, in which the nucleation of MoS_2_ and WS_2_ are along the <10$\bar{1}$0> step edges of sapphire, further proving the effect of step edges on the alignment of TMDs domains. However, the direction of the step edge on sapphire is highly dependent on the miscut direction, and it is rather difficult to maintain a fixed cutting angle with such a high accuracy (e.g.,1°) over an inch scale.

Herein, we design a substrate-step templated growth strategy for synthesizing large-area uniform, unidirectionally aligned, monolayer single-crystal TMDs ribbons, by using the step edges of vicinal Au(111) single crystals as growth fronts. The superiorities of this route are summarized as follows: (1) the uniformly oriented step edges on high-index Au facets can trigger the anisotropic growth of TMDs, and direct the alignment of the resulted monolayer ribbons; (2) the chemical inertness of Au substrate to chalcogen precursor makes it a universal template for synthesizing various monolayer TMDs ribbons (e.g., MoS_2_, WS_2_, MoSe_2_, WSe_2_); (3) different from the growth on insulating substrates, the monolayer TMDs grown on Au metals are featured with relatively strong interface coupling, which can be another parameter for mediating the van der Waals epitaxial growth of 2D layered materials toward wafer-scale single crystals; (4) the synergistic effect of substrate-step-edge guided 1D epitaxy, combined with substrate-lattice-match directed 2D epitaxy modes are expected to direct the epitaxial growth of single-crystal TMDs monolayers. This work is expected to offer an alternative strategy for the synthesis of monolayer patterned TMDs ribbons or wafer-scale single-crystal films. The practical applications of the dimension controllable monolayer materials (ribbons or films) will also be demonstrated in more versatile fields, e.g., as channel materials in high-performance electronic devices and as catalysts in HER.

## Results

### Theoretical calculations for the evolution of monolayer TMDs ribbons

To initialize the growth of monolayer MoS_2_ ribbons, it is a general route to introduce anisotropic template with broken symmetry, e.g., by introducing substrate steps^27^. Such a substrate-template-directed synthesis strategy has previously been used for the growth of 1D GaN^28^, graphene^29^, and MoS_2_ nanowires^30^. In our experiment, a series of high-miller-index Au facets vicinal to (111) with bunched atomic steps were selected as growth templates, as obtained by melting and resolidifying Au foils on W templates (see Methods for more details). As reported previously, the types of the MoS_2_ terminated edges were quite different for 1D stripes and 2D triangles, i.e., Mo-zigzag (Mo-zz)/S-zigzag (S-zz) edges and Mo-zz edges, respectively, as characterized by transmission electron microscope (TEM) in previous literatures^27,28^. Moreover, the edge type of monolayer MoS_2_ achieved by the CVD growth process highly depended on the S/Mo ratio of precursors^31–34^. In this regard, the effect of S/Mo ratio should have a significant effect on the morphology of monolayer MoS_2_.

**Fig. 1:**
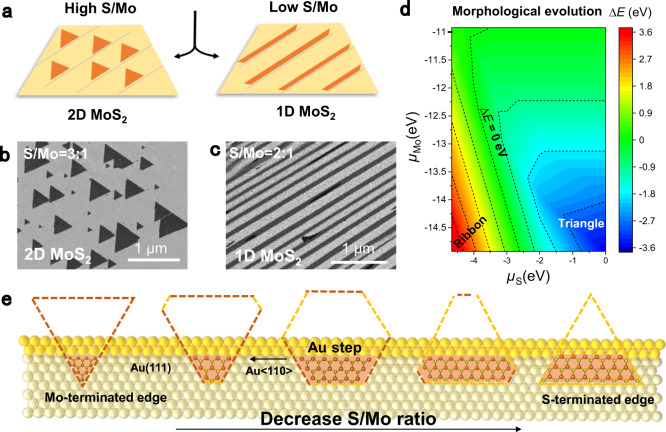
Morphological evolution of monolayer MoS_2_ from triangular to ribbon-like shaped domains. **a** Schematic illustration of the morphological evolution of MoS_2_. **b**, **c** SEM images of 2D monolayer MoS_2_ triangles (**b**) and 1D ribbons (**c**) achieved at different S/Mo ratios. **d** Nucleation barrier (Δ*E*) difference mapping between S-zz and Mo’-zz edges under different chemical potentials of Mo (*μ*_Mo_) and S (*μ*_S_). For the case of high *μ*_S_ and low *μ*_Mo_ (bottom right region), the nucleation barrier of S-zz is smaller than that of Mo’-zz edge (Δ*E* < 0), Mo’-zz edge is the dominating edge and the resulted MoS_2_ flake presents a triangle shape. As *μ*_S_ decreases (Δ*E* > 0, bottom left region), the S-zz edge becomes the dominating edge and the resulted MoS_2_ flake shows quasi ribbon-like shape. **e** Schematic view showing the morphology variations of monolayer MoS_2_ islands with decreasing the S/Mo ratio. Orange and yellow spheres indicate Mo and S atoms, while the orange and yellow lines represent Mo and S terminated edges, respectively.

To confirm this, CVD growth was executed on a representative Au(223) facet with MoO_3_ and S as precursors (growth conditions: 750 °C for 3 min, Supplementary Fig. 1). When the mass ratio of S/Mo was set at 3:1, monolayer MoS_2_ triangular domains were obtained (Fig. 1b). As the S/Mo ratio decreased to 2:1, monolayer MoS_2_ ribbons evolved under similar growth conditions (Fig. 1c). X-ray photoemission spectroscopy (XPS) spectra confirmed the formation of MoS_2_ compounds (Supplementary Fig. 2). Atomic force microscope (AFM) images of as-grown monolayer MoS_2_ triangular domains and ribbons on Au substrates (Supplementary Fig. 3) clearly show that the edges of MoS_2_ are usually aligned along the steps of Au substrates, providing straightforward evidence for the step-edge-guided growth mechanism. Nevertheless, further decreasing the S/Mo ratio to 1:1 led to the formation of irregularly shaped MoO_*x*_S_2−*x*_ crystals (Supplementary Fig. 4), probably due to the insufficient feeding of the S feedstock^30^.

To further understand the role of S/Mo ratio in the morphological evolution of monolayer MoS_2_, density functional theory (DFT) calculations were then performed to simulate the MoS_2_ growth on Au(111). The kinetic growth of MoS_2_ can be analyzed by exploring the nucleation barriers of two dominating edges on Au(111) around <110> step during the atomic accretion process, i.e., S-zz and reconstructed Mo-terminated zigzag (Mo’-zz) edges due to their higher nucleation barriers than those of armchair, and Mo-zz (see details in Supplementary Figs. 5‒7). Using Mo atoms and S_2_ dimers as the feeding units, the relative nucleation barriers (Δ*E*) for these two edges under different chemical potentials of Mo and S (labeled as *μ*_Mo_ and *μ*_S_, respectively) were calculated. For the case of high *μ*_S_ and low *μ*_Mo_ (bottom right in Fig. 1d), the nucleation barriers of S-zz are smaller than that of Mo’-zz edges (Δ*E* < 0). Hereby, Mo-oriented edges should be prevalent in parallel with the evolution of triangular-shaped domains. As *μ*_S_ decreases (Δ*E* > 0, bottom left in Fig. 1d), the growth velocity of S-zz edge declines significantly, and S-zz edge becomes the dominating one. Following our previous results^20^, the minimum energy configuration corresponds to Mo-zz edges docking to Au <110> steps on the (111) surface. Thereby, the ribbons terminated with Mo-zz edges along the Au <110> steps (on one side) and terminated with free S-zz edges on the terraces (on the other side) should be more preferentially evolved. The schematic diagram in Fig. 1e depicts the shape evolutions of MoS_2_ from triangles to isosceles trapezoid or quasi stripes with the change of S/Mo ratios.

### Controllable growth of uniformly aligned monolayer TMDs ribbons

**Fig. 2:**
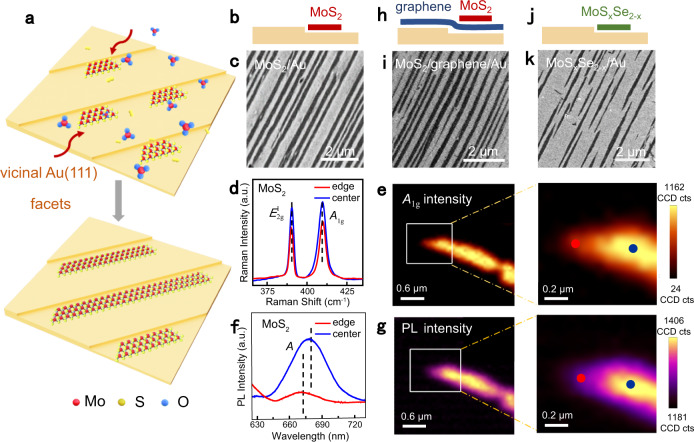
Universal growth of aligned monolayer TMDs ribbons along the step edges of Au(111) vicinal facets. **a** Schematic illustration of the growth of well-aligned monolayer MoS_2_ ribbons along the step edges of vicinal Au(111) facets. The red, yellow, and blue spheres represent Mo, S, and O atoms, respectively, and the red arrows indicate the diffusion pathways of the active species. **b**, **c** Schematic side view and SEM image of aligned monolayer MoS_2_ ribbons grown on Au(223) facet, respectively. **d** Representative Raman spectra of the edges (red) and centers (blue) of monolayer MoS_2_ ribbons transferred on SiO_2_/Si substrates. **e** Raman mapping on the intensity of *A*_1g_ peak for a MoS_2_ ribbon. The right panel is the magnified image of the white rectangle marked region in the left panel. The red and blue dots mark the edge and center positions of MoS_2_ ribbon, respectively. a.u., arbitrary units. **f**, **g** Representative PL spectra of the edges (red) and centers (blue) of a MoS_2_ ribbon and its PL mapping on the intensity of A peak. The right panel is the magnified image of the white rectangle marked region in the left panel. The red and blue dots in (**g**) mark the edge and center positions of MoS_2_ ribbon, respectively. **h**, **i** Schematic side view and SEM image of monolayer MoS_2_ ribbons grown on monolayer graphene covered Au(213) facet, respectively. **j**, **k** Schematic side view and SEM image of monolayer MoS_x_Se_2-x_ ribbons grown along the steps of Au(235) facet, respectively.

According to our theoretical calculations, the following mechanism is proposed for the formation of unidirectionally oriented monolayer MoS_2_ ribbons. As schematically illustrated in Fig. 2a, MoS_2_ species tend to first nucleate at the step edges on the high-miller-index Au facets, considering of the high binding energy between them. Under a relatively small S/Mo ratio, the growth rate of S-zz edge was slower than that of Mo-zz edge, inducing the formation of MoS_2_ ribbons terminated by Mo-zz and S-zz edges on either side.

As evidenced by scanning electron microscopy (SEM) images (Fig. 2c), the derived MoS_2_ ribbons are uniformly distributed on the Au(223) facet with unidirectional alignment. These monolayer MoS_2_ ribbons present straight edges, totally distinct from the sawtooth-like edged nanoribbons stringed by triangular domains (grown on β-Ga_2_O_3_ (100))^13^. Moreover, the widths and lengths of the obtained MoS_2_ ribbons are tunable within 20‒120 nm and 3‒30 um, respectively (Supplementary Fig. 8). Their aspect ratios (10^2^‒10^3^) are much larger than most of the TMDs ribbons reported previously (10‒10^2^)^11–13,30,31,35,36^ (Supplementary Table 1). AFM characterization of a typical MoS_2_ ribbon shows a thickness of ~0.7 nm, again confirming its monolayer nature (Supplementary Fig. 9).

Raman and photoluminescence (PL) spectra were then collected from the monolayer MoS_2_ ribbons transferred on SiO_2_/Si substrates. The Raman characteristic peaks are fixed at ~386 cm^−1^ (*E*^1^_2g_) and 406 cm^−1^ (*A*_1g_) (Fig. 2d), with a frequency difference (Δ) ~20 cm^−1^, justifying the monolayer nature of the MoS_2_ ribbons^37^. However, the Raman intensity of the ribbon edge is slightly lower than that of the center region (Fig. 2e), probably due to the reduced electron density at the ribbon edge. Meanwhile, the PL spectrum from the ribbon edge exhibits a blue-shifted peak (at ~677 nm) and lower intensity (Fig. 2g) relative to the center region (at ~684 nm), which may arise from the strain at the ribbon edge^38,39^.

More intriguingly, such step-edge-guided growth of monolayer MoS_2_ ribbons was also realized on monolayer graphene or *h*-BN-covered vicinal Au(111) facets. As shown in Fig. 2i, well-aligned monolayer MoS_2_ ribbons occurred on a monolayer graphene-coated Au(213) facet, as confirmed by the coexistence of characteristic Raman signals for monolayer MoS_2_, i.e., *E* ^1^_2g_ (~386 cm^−1^) and *A*_1g_ (~406 cm^−1^) peaks, and graphene on the same region (Supplementary Fig. 10). Besides, patterned monolayer MoS_2_ ribbons were also achieved on the monolayer *h*-BN/Au(111) template (Supplementary Fig. 11). The compatibility of Au substrate with the growth of TMDs, graphene, and *h*-BN makes it possible to construct van der Waals heterostructures based on these 2D layered materials.

Apart from monolayer MoS_2_, monolayer TMDs alloys ribbons, such as MoS_*x*_Se_2–*x*_, were also achieved on an Au(235) facet by using mixed S and Se as chalcogen precursor (Fig. 2k). Raman spectrum (inset of Fig. 6g) shows two groups of characteristic peaks, in line with that of MoS_2_ (at ~386 and 405 cm^−1^) and MoSe_2_ (at ~270 cm^−1^), justifying the formation of MoS_*x*_Se_2–*x*_ compounds^40^. Given these facts, the substrate-step-edge guided growth route can be utilized as a universal pathway for the growth of TMDs ribbons and their alloys.

### Preparation of inch-scale high-miller-index Au single-crystal films

**Fig. 3:**
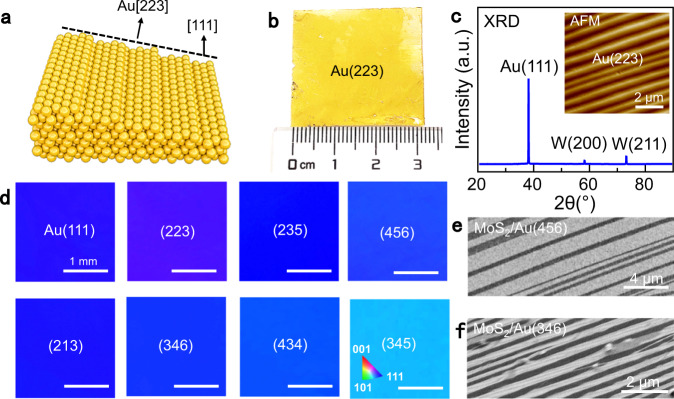
Preparation of inch-scale high-miller-index Au single-crystal films (Au(111) vicinal facets). **a** Schematic illustration of a high-index Au(223) facet. **b** Representative photography of the evolved Au(223) single-crystal film on the W template. **c**. XRD pattern of Au/W foils showing the evolution of single-crystal Au(111) film. Inset: AFM image of a single-crystal Au film with Au(223) facet. **d** Representative EBSD IPF maps of the as-prepared Au films, with crystal facets of (111), (223), (235), (456), (213), (346), (434), (345), respectively. The scale bars in all panels are 1 mm. **e**, **f** SEM images of the unidirectionally aligned monolayer MoS_2_ ribbons evolved on single-crystal Au(456) (**e**) and Au(346) (**f**), respectively.

The key point of this synthetic route relies on the preparation of anisotropic substrate of vicinal Au(111) single-crystal facets. To achieve this, Au foils on W templates were melted and resolidified at ~1100 °C and maintained for ~20 min. The schematic and photograph of a representative Au film (with a size of 3 cm × 3 cm) featured with Au(223) facet are presented in Fig. 3a, b, respectively. The facet surface was characterized by uniformly distributed, parallel bunched steps, as evidenced by the AFM image in Fig. 3c. X-ray diffraction (XRD) pattern of the as-prepared Au/W film shows the (111) peaks of Au film, and the (200) and (211) peaks of W foil (Fig. 3c).

However, due to the limited resolution of XRD, it is difficult to distinguish the exact miller-index of the Au facet. Further electron backscatter diffraction (EBSD) characterization reveals the evolution of such a single-crystal Au(223) facet (Fig. 3d). Large-area EBSD maps collected on the whole sample surface show uniform color contrast and nearly undetectable angular variation (Supplementary Fig. 12), confirming the single crystallinity of the Au(223) facet. Notably, a series of single-crystal Au(111) vicinal facets were also achieved through the current annealing process, including (235), (213), (346), (456), etc. (Fig. 3d and Supplementary Fig. 13), as defined by the homogeneous color contrasts in the inverse pole figure maps.

The derived high-miller-index Au facets belong to a category of facets vicinal to Au(111), which are composed of a regular succession of (111) terraces separated by monatomic steps. Notably, the appearance of a specific high-index facet is relatively random, as similarly proposed in the preparation of atomic sawtooth high-miller-index Au facets through different annealing processes^21^. Herein, the formation of the high-index facet is probably due to the close contact between Au liquid and W template at high temperature, in which the strain energy should be the driving force, rather than the surface energy for the formation of Au(111). This phenomenon was also reported in the preparation of high-miller-index single-crystal Cu foils, in which the stress on Cu foil was introduced by using graphite susceptor^41^. Notably, a variety of vicinal Au(111) facets, independent of the specific index (e.g., Au(456), Au(346)), were all proved to be capable of inducing the formation of monolayer MoS_2_ ribbon arrays (Fig. 3e, f). Besides, the as-grown monolayer MoS_2_ ribbon presents a relatively high density on the high-miller-index Au facet with a high density of step (Supplementary Fig. 14). This provides an effective route for ribbon density regulation.

### Monolayer TMDs single-crystal films merged by 1D ribbons

**Fig. 4:**
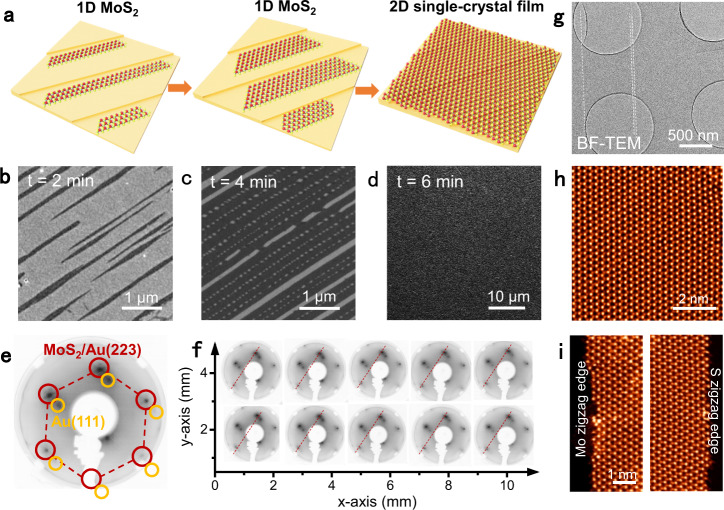
Monolayer single-crystal MoS_2_ films achieved by the growth and merging of monolayer MoS_2_ ribbons. **a** Schematic illustration regarding the growth of monolayer MoS_2_ single-crystal film through the merging of MoS_2_ ribbons. Red and yellow spheres represent Mo and S atoms, respectively. **b**–**d** SEM images showing the growth process from narrow MoS_2_ ribbons (**b**), wide ribbons (**c**), to continuous 2D film (**d**) by extending the growth time (t) from ∼2, ∼4, to ∼6 min, respectively. **e** Representative LEED pattern of monolayer MoS_2_ single-crystal on the Au(223) facet. The spots indicated by red circles show the moiré satellite peaks for MoS_2_/Au(223). The spots indicated by yellow circles arise from the Au(111) facet. **f** More LEED patterns collected over an area of 10 mm × 4 mm. The red lines in the same direction indicate the same orientation of these patterns. **g** Low-magnification bright-field TEM image of a nearly full-coverage MoS_2_ film transferred on the TEM grid. **h** Atomically resolved STEM image of the monolayer MoS_2_ film, confirming its defect-free feature. **i** STEM images captured from the left and right edges of a monolayer MoS_2_ ribbon presenting Mo-zz and S-zz edges, respectively.

The lateral growth and merging behavior of monolayer TMDs ribbons were then investigated by deliberately increasing the coverage. As shown in Fig. 4b‒d, upon extending the growth time from 2, 4 to 6 min, monolayer MoS_2_ ribbons with average widths of ~130 and ~190 nm, and continuous full-coverage MoS_2_ film were achieved on the Au(223) facet steadily. In this regard, the growth should obey a step-flow growth mode, inducing the formation of single-crystal film through the merging of unidirectionally aligned grains. The single crystallinity of the monolayer film was first verified by low-energy electron diffraction (LEED) measurements. The obtained diffraction spots indicate the existence of MoS_2_ on the high-index Au(223) stepped surface (indicated by red circles in Fig. 4e) and Au(111) terraced substrate (yellow circles). The uniform diffraction pattern that appeared on the inch-scale sample should indicate the epitaxial growth of monolayer MoS_2_ single crystal on the Au(223) facet (Fig. 4f and Supplementary Fig. 15).

TEM and scanning transmission electron microscopy (STEM) characterizations were then conducted to identify the lattice orientation and edge structure. A representative atomically resolved STEM image presents well-ordered honeycomb lattices (Fig. 4h). On large scales, the crystal lattice of MoS_2_ maintains the same orientation, showing almost no obvious grain boundary. Selected-area electron diffraction (SAED) patterns collected on the film (Supplementary Fig. 16) reveal nearly identical lattice orientation (deviation smaller than ±0.1°), highly indicative of the single-crystal nature of the MoS_2_ film. Moreover, the edge types on both sides of the monolayer MoS_2_ ribbon are identified as Mo-zz and S-zz edges from both SAED pattern (Supplementary Fig. 17) and atomic-resolution STEM image (Fig. 4i), and this result is consistent with our previous calculations. Dark-field TEM images of the monolayer MoS_2_ film show uniform intensity over the entire area, again confirming its single crystallinity (Supplementary Fig. 18).

**Fig. 5:**
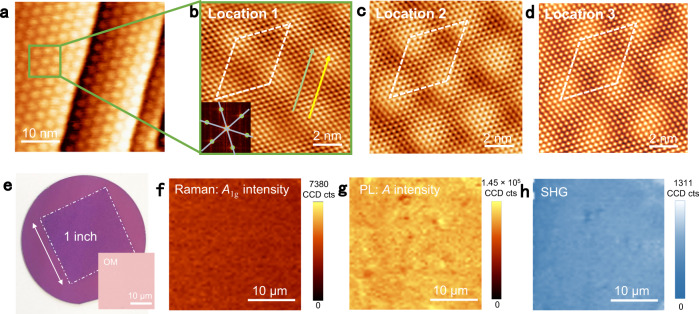
Single-crystal property characterizations of the continuous monolayer MoS_2_ film achieved by the growth and merging of monolayer MoS_2_ ribbons. **a** Large-area STM image (*V*_Tip_ = –0.61 V, *I*_Tip_ = 3.55 nA) of the monolayer MoS_2_ film on the Au(223) facet with Au(111) terrace. **b** Magnified STM image (–0.1 V, 30.77 nA) of the green square region in (**a**), presenting the perfect alignment of MoS_2_ lattice direction with moiré row direction, as denoted by green and yellow arrows, respectively. Inset: corresponding 2D-FFT pattern. **c**, **d** Representative atomically resolved STM images of the moiré pattern for MoS_2_/Au(111), presenting a fixed period of ∼3.21 ± 0.10 nm (marked by white rhombus; −0.10 V, 40.95 nA; −0.1 V, 32.79 nA). **e** Photograph and corresponding OM image of a 1-inch single-crystal MoS_2_ film transferred on SiO_2_/Si. **f** Raman mapping on the *A*_1g_ peak intensity for the monolayer MoS_2_ single crystal showing a uniform color contrast. **g** PL mapping on the *A* exciton intensity of the monolayer film. **h** SHG mapping of the monolayer MoS_2_ single crystal.

On-site scanning tunneling microscopy (STM) measurements were then performed to determine the crystalline quality. A large-area STM image reveals the covering of a continuous monolayer MoS_2_ film on the Au(111) terraces (Fig. 5a). Further magnified image (from the region marked by the green square in Fig. 5a) presents the evolution of a large-area hexagonally shaped moiré pattern with a period of ∼3.21 ± 0.10 nm (Fig. 5b). Moreover, the orientation of the MoS_2_ atomic row aligns well with the moiré row direction (as denoted by green and yellow arrows, respectively). This alignment was also confirmed by the corresponding 2D fast Fourier transform image (inset of Fig. 5b), indicating an epitaxial relationship between them. As calculated, the moiré supercell (highlighted by a white rhombus) is in line with a (10 × 10) supercell of MoS_2_ (*a*_1_ = 0.320 nm) on an (11 × 11) supercell of Au(111) (*a*_2_ = 0.288 nm). Furthermore, atomic-resolution STM images collected from the entire film show the same lattice orientation (Fig. 5b‒d and Supplementary Fig. 19), highly indicating the single-crystal nature of the monolayer MoS_2_ film.

Further photograph and optical microscopy (OM) images reveal homogenous color contrasts for the 1-inch single-crystal MoS_2_ film transferred on SiO_2_/Si (Fig. 5e). Additional Raman and PL intensity mappings also suggest the homogeneous optical properties (Fig. 5f, g). Moreover, almost no obvious contrast difference can be noticed in the second-harmonic generation intensity mapping (Fig. 5h), which unambiguously confirms the single crystallinity of the monolayer MoS_2_ film.

### Syntheses of other monolayer TMDs ribbon arrays

**Fig. 6:**
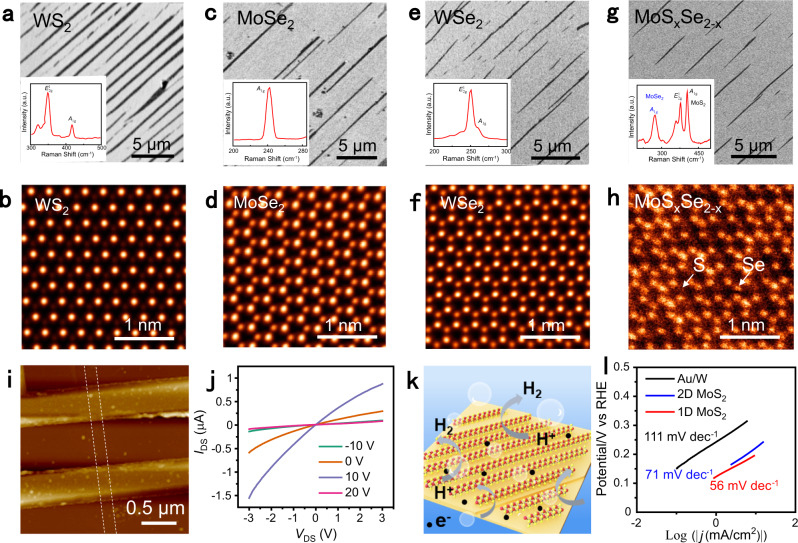
Au-step-edge-directed growth of other monolayer TMDs ribbons. **a**, **b**. SEM image (**a**), representative Raman spectrum (inset of (**a**)), and STEM image (**b**) of as-grown monolayer WS_2_ ribbon arrays. **c**, **d** SEM image (**c**), representative Raman spectrum (inset of (**c**)), and STEM image (**d**) of as-grown MoSe_2_ ribbon arrays. **e**, **f**. SEM image (**e**), representative Raman spectrum (inset of (**e**)), and STEM image (**f**) of as-grown monolayer WSe_2_ ribbon arrays. **g**, **h** SEM image (**g**), representative Raman spectrum (inset of (**g**)), and STEM image (**h**) of as-grown monolayer MoS_x_Se_2-x_ ribbon arrays. **i** AFM image of a representative monolayer MoS_2_ ribbon-based device with a width of ~110 nm. **j** Output characteristics of the monolayer MoS_2_ ribbon-based device. **k** Schematic illustration of the HER process. Red and yellow spheres represent Mo and S atoms, respectively, and the black dots denote the electric charges. **l** Tafel plots of the as-grown monolayer MoS_2_ triangles and monolayer MoS_2_ ribbons on Au/W electrodes.

To further display the universality of our synthetic strategy, other TMDs ribbons and alloys, such as WS_2_, MoSe_2_, WSe_2_, and MoS_x_Se_2-x_ were also synthesized on the high-miller-index facets vicinal to Au(111) (Fig. 6a‒h), by using MO_3_ (M = Mo, W) and S, Se as precursors. Corresponding Raman data and STEM image confirm the desired chemical composition and monolayer nature of the ribbons.

The ready access to monolayer TMDs ribbons provides us an opportunity for exploring their exotic electronic and catalytic performances. In order to characterize their electronic properties, back-gated FETs were constructed for MoS_2_ ribbons on SiO_2_/Si substrates (Fig. 6i). The transfer (Supplementary Fig. 20) and output curves (Fig. 6j) of a representative device show typical n-type transfer characteristics. Statistical data measured on these FETs (Supplementary Fig. 21) show a narrow electron mobility distribution 7‒11 cm^2^ V^−1^ s^−1^ and an on/off ratio of 10^5^‒10^6^, comparable to the performance of 2D MoS_2_ monolayers achieved on Au substrates^20^. Notably, the performance of the FETs based on monolayer MoS_2_ grown on Au are not as good as those of monolayer MoS_2_ films grown on sapphire^16,28,42^. This may originate from the different sample transfer processes used for them (e.g., electrochemical bubbling transfer), wherein contamination and structural defects were usually introduced. Hereby, a more efficient and clean sample transfer process should be developed in the future.

Besides the potential application as channel material, monolayer MoS_2_ ribbon was also predicted to possess higher catalytic performance in HER than its 2D counterpart, owing to the exposure of more abundant active edge sites. The applications of 1D monolayer MoS_2_ ribbons and 2D monolayer MoS_2_ flakes grown on Au/W substrates are also addressed as electrocatalysts in HER (Fig. 6k). The overpotential (at a current density of 10 mA cm^−2^) and Tafel slope of monolayer MoS_2_ ribbons were measured to be ~194 mV and 56 mV dec^−1^, respectively, obviously lower than the values for 2D MoS_2_ flakes (~220 mV and 71 mV dec^−1^) (Fig. 6l and Supplementary Fig. 22). Moreover, the catalytic current density exhibited negligible loss (<5%) after 1000 cycles, indicating the relatively high catalytic stability. The loading of the catalysts can be further increased toward low-cost, high-efficiency, and large-scale application explorations.

## Discussion

In conclusion, we have realized the direct synthesis of periodic monolayer TMDs ribbon arrays, and wafer-scale TMDs single crystals on high-miller-index Au facets vicinal to (111) via a designed step-edge-directed synthetic strategy. Monolayer TMDs ribbons characterized by strictly monolayer thickness, high aspect ratio, relatively straight edge, and uniform alignment have been achieved. Based on this, an alternative epitaxial growth approach for wafer-scale TMDs single crystals is proposed by merging these uniformly aligned monolayer ribbons through a unique 1D edge-epitaxy mode. This work hereby opens a pathway for the single-crystal growth of 2D semiconductors on a wafer scale, as well as deepens our understanding of the epitaxial growth mechanism for the wafer-scale synthesis of 2D layered materials on insulating or conducting templates. This work should also propel the application explorations of monolayer TMDs ribbons or single crystals as channel materials in nanoelectronics and efficient catalysts in energy-related fields.

## Methods

### Preparation of vicinal Au(111) single crystals

The commercial polycrystalline gold foils (99.99%, ~50 µm thickness) were first ultrasonic cleaned in HCl solution (20% wt) for 10 min, and then in an acetone solution for 10 min. To prepare the high-miller-index facets vicinal to Au(111), a piece of cleansed gold foil was placed on an etched tungsten foil on a graphite boat, annealing at ~1100 °C for 20 min with a mixture of Ar/H_2_ (300/50 sccm) as the carrier gas. The gold will firstly melt into liquid at ~1100 °C, and resolidified to induce the formation of Au(111) vicinal facets after cooling down processes.

### Synthesis of monolayer TMDs ribbons

For the growth of MoS_2_ ribbons, the MoO_3_ and S powders were placed in the upstream of Au(111)/W substrate at a distance of 4 and 20 cm in a three-zone tube furnace, respectively. The growth was performed in a low-pressure environment with the protection of Ar (50 sccm) gas. The typical temperatures of S, MoO_3_ (2‒3 mg), and Au(111) substrates were ~100, ~530, and ~750 °C, respectively. The S/Mo ratio was defined by the quantity of S and MoO_3_ precursors. Notably, this growth temperature was relatively higher than that used for the growth of triangular monolayer MoS_2_ domains (~720 °C) in our previous work^20^.

For the growth of monolayer WS_2_ ribbons, the typical temperatures of S, WO_3_, and Au substrates were set at ~100, 820, and 820 °C, respectively. For the growth of 1D monolayer MoSe_2_ and WSe_2_ ribbons, a mixture of Ar (50 sccm) and H_2_ (5 sccm) was used as the carrier gas. The Se powder was placed in the upstream of a substrate at a distance of 16 cm. The typical temperatures for Se, MoO_3_ (WO_3_), and Au substrates were set at ~200, 530 (820), 820 °C, respectively. For the synthesis of monolayer MoS_*x*_Se_2–*x*_ ribbons, the MoO_3_ precursor and a mixed S and Se precursor were placed in the upstream of Au/W substrate at a distance of 1 and 20 cm, respectively. The typical temperatures for the Au substrates, MoO_3_, and S/Se mixture were set at 850, 800, and 200 °C, respectively. The growth process was performed under a mixture flow of Ar (50 sccm) and H_2_ (5 sccm) gases. The synthetic recipes for MoS_2_ ribbons grown on graphene and *h*-BN monolayers are provided in the Supplementary information.

### Calculation methods

The theoretical calculations based on DFT were performed by the Vienna ab initio simulation package^43^. Details of calculations are provided in the Supplementary information.

### Characterization of MoS_2_ ribbons

The MoS_2_ ribbons and single crystals were systematically characterized by SEM (Hitachi S-4800, 2 kV), OM (Nikon ECLIPSE, LV100ND), Raman and PL spectroscopy (Horiba Jobin-Yvon, LabRAM HR800, with an excitation wavelength of 532 nm), spectral imaging (WITec, Alpha 300R), AFM (Bruker, Dimension Icon), XPS (Kratos Analytical Ltd. AXIS-Ultra with monochromatic Al Ka X-ray), TEM (FEI Tecnai F20, acceleration voltage of 200 kV), STEM (JEOL ARM200F, acceleration voltage 80 kV), and STM (Omicron, the base pressure: 10^−10^ mbar). The LEED measurements were carried out in an ultra-high vacuum (3 × 10^−10^ Torr) and the electron energy was fixed at 96 eV. The electron beam spot size is about 500 μm. The single-crystal Au(111) vicinal facets were characterized by XRD (Bruker D8-Discover, with a Cu Kα radiation source) and EBSD (Oxford instruments).

## Supplementary information

Supplementary Information

## Data Availability

The data that support the findings of this study are available from the corresponding author upon request.
