# Supplementary material for: Epitaxial growth of inch-scale single-crystal transition metal dichalcogenides through the patching of unidirectionally orientated ribbons
Source: Nat Commun. 2022 Jun 10;13:3238. doi: 10.1038/s41467-022-30900-9 (PMC9187673; doi:10.1038/s41467-022-30900-9)
Supplement: Supplementary file 1 — Supplementary Information [file 41467_2022_30900_MOESM1_ESM.docx]

**Epitaxial growth of inch-scale single-crystal transition metal dichalcogenides through the patching of unidirectionally orientated ribbons**

Pengfei Yang^1#^, Dashuai Wang^2^^#^, Xiaoxu Zhao^1,3#^, Wenzhi Quan^1^, Qi Jiang^4^, Xuan Li^5^, Bin Tang^5^, Jingyi Hu^1^, Lijie Zhu^1^, Shuangyuan Pan^1^, Yuping Shi^1^, Yahuan Huan^1^, Fangfang Cui^1^, Shan Qiao^4^, Qing Chen^6^, Zheng Liu^3^, Xiaolong Zou^2*^, and Yanfeng Zhang^1*^

**Contents**

**I. Supplementary Methods**

1. Calculation Methods.

2. Synthesis of 1D MoS_2_/2D graphene and 1D MoS_2_/2D h-BN heterostructures.

3. Device fabrications and electrical property measurements.

**II. Supplementary Figures and Tables**

Supplementary Fig. 1 Schematic illustration of the CVD growth process of MoS_2_ ribbons.

Supplementary Fig. 2 XPS spectra of as-grown MoS_2_ ribbons grown on Au(223) facet.

Supplementary Fig. 3 AFM images of as-grown monolayer MoS_2_ triangular domains and ribbons on Au substrates.

Supplementary Fig. 4 SEM images of MoO_x_S_2−x_ crystals under the S/Mo ratio of 1:1.

Supplementary Fig. 5 Four kinds of edge configurations of MoS_2_ ribbons.

Supplementary Fig. 6 The diffusion barriers and paths of S and S2 dimmer on Au (111) plane and Au <110> steps.

Supplementary Fig. 7 The dependence of nucleation barriers of four edges of MoS_2_ ribbons (S-zz, Mo-zz, AC, and Mo’-zz) as a function of *µ*_Mo_ at different *µ*_S_.

Supplementary Fig. 8 Large-area aligned MoS_2_ ribbon arrays grown on Au(223) facet.

Supplementary Fig. 9 AFM image of a MoS_2_ ribbon transferred on SiO_2_/Si substrate.

Supplementary Fig. 10 Monolayer graphene films and MoS_2_/graphene vertical stacks grown on Au(213) facet.

Supplementary Fig. 11 MoS_2_ ribbon arrays grown on monolayer *h*-BN coated-Au(111).

Supplementary Fig. 12 EBSD maps in the normal direction (ND) of the as-prepared Au(223)/W substrate, collected at six randomly selected positions.

Supplementary Fig. 13 Representative EBSD IPF maps of as-prepared vicinal Au(111) facets.

Supplementary Fig. 14 Morphologies of monolayer MoS_2_ ribbons grown on different high-miller-index Au facets.

Supplementary Fig. 15 Representative LEED patterns from twelve randomly selected regions of the single-crystal MoS_2_ film grown on Au(223).

Supplementary Fig. 16 TEM characterizations of a nearly continuous MoS_2_ film.

Supplementary Fig. 17 Characterization of the crystal structure of monolayer MoS_2_ ribbon by electron diffraction.

Supplementary Fig. 18 DF-TEM characterizations of a coalesced monolayer MoS_2_ film.

Supplementary Fig. 19 STM images from several randomly selected locations on the as-grown single-crystal MoS_2_ film.

Supplementary Fig. 20 Electrical performance of a representative monolayer MoS_2_ ribbon-based FET.

Supplementary Fig. 21 Statistical analysis of the electrical performances for monolayer MoS_2_ ribbon-based FETs devices.

Supplementary Fig. 22 Electrocatalytic HER performances of as-grown MoS_2_ ribbons on Au/W.

Supplementary Table 1 Comparison of the aspect ratios of TMDs ribbons/channels reported in the literatures with our samples.

**I. Supplementary Methods**

**1. Calculation Methods**. The theoretical calculations based on DFT were performed by the Vienna ab initio simulation package. The projector augmented wave potential was used with a plane-wave energy cutoﬀ of 400 eV. The exchange correlation energy was described by the generalized gradient approximation proposed by Perdew, Burke, and Ernzerhof^1^. For geometry optimization, the Brillouin-zone integration was performed using 1×1×1 Gamma-centered grid for k-point sampling. Geometry optimizations were carried out with the convergence threshold of 10^−4^ eV per atom in energy and 0.1 eV∙Å^−1^ in force. To deal with the van der Waals forces, the Tkatchenko-Scheﬄer method was adopted in the calculations^2^. For the kinetic models of MoS_2_ growth on Au, the 3-layer Au slab with <110> step was constructed, with the periodic step length of 28.8 Å to match the zigzag edges of MoS_2_ ribbons docking to <110> steps following the results in ref [9]. The lattice length along the other direction is 30.3 Å. The initial widths of MoS_2_ ribbon terminated with zigzag and armchair edges were set ~9.4 and 11.2 Å, respectively. While zigzag ribbons were periodic along step direction, the two zigzag ends of armchair ribbons were docked to the step (Supplementary Fig.1). During optimization, the bottom layer of the Au slab was ﬁxed, whereas the MoS_2_ nanoribbon and top two Au layers were relaxed. To avoid spurious interaction between images from periodic boundary conditions, a vacuum separation between two neighboring slabs was set to 20 Å. Furthermore, for diffusion of S single atoms and S2 dimer on the Au (111) surface and along <110> step, the climbing-image nudged elastic band method was used with eight images, including the initial and final positions. The nucleation barrier is calculated as $E_{f}= E_{\mathrm{tot}}-E_{\mathrm{sub}}-{N_{\mathrm{Mo}}\mu}_{\mathrm{Mo}}-{N_{s}\mu}_{S}$, where *E*_tot_ and *E*_sub_ are the total energies of the monolayer MoS_2_ edges on the Au substrate with and without adding Mo/S atoms, respectively, and *N*_Mo/S_ is the number of added Mo or S atoms. For simplicity, the chemical potentials of Mo and S are restricted by $\mu_{\mathrm{Mo}}+2\mu_{S}=\mu_{\mathrm{MoS}_{2}}$ following quasi-equilibrium conditions, where $\mu_{\mathrm{MoS}_{2}}$ is the chemical potential of a MoS_2_ unit in the single-layer 2H phase. In general, for solid or liquid state Mo, *µ*_Mo_ changes slightly; for gas-state S, *µ*_S_ is significantly influenced by pressure, volume and concentration, and can be easily calculated following the standard statistics mechanics^3^. Therefore, high S/Mo ratio is proposed to correspond to high *µ*_S_, and relatively low *µ*_Mo_, or vice versa.

**2. Synthesis of** **1D MoS_2_/2D graphene and 1D MoS_2_/2D *h*-BN heterostructures**. A two-step CVD route was used for the synthesis of 1D-MoS_2_/2D-graphene and 1D-MoS_2_/2D-*h*-BN heterostructures. For the synthesis of monolayer graphene films, the substrate was first heated from room temperature to ~990 °C, with 200 sccm Ar and 30 sccm H_2_ as carrier gases. CH_4_ (3 sccm) precursor was introduced to the furnace for 3h to grow full-covered graphene monolayer. For the synthesis of 2D *h*-BN monolayers, ammonia borane was used as the *h*-BN precursor. The substrate was heated to ~1030 °C and maintained for ~60 min with the protection of Ar (300 sccm) and H_2_ (50 sccm) mixed gas.Then, monolayer MoS_2_ ribbons was then deposited on monolayer graphene and *h*-BN film using the same method mentioned above.

**3. Device fabrications and electrical property measurements**. Back-gated FETs based on monolayer MoS_2_ ribbons were constructed on SiO_2_/Si substrates with an oxide layer of ~300 nm. The devices were fabricated by electron-beam lithography and electronbeam deposition of Ti/Au (5/60 nm). The electrical properties were measured with a probe station (Lake Shore, Model TTP4) in a vacuum chamber (<1.3 mTorr) at room temperature, by using a semiconductor characterization system (Keithley 4200-SCS).

**II. Supplementary Figures and Tables**

**
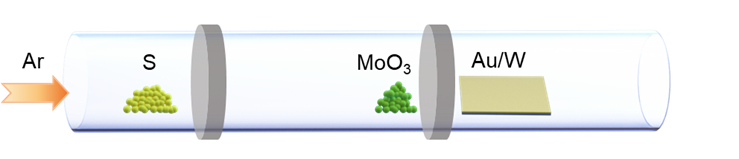
**

**Supplementary Fig. 1.** Schematic illustration of the CVD growth process of MoS_2_ ribbons. The uniformly aligned monolayer MoS_2_ ribbons were grown using a three-zone furnace. The MoO_3_ and sulfur powder precursors were placed at the upstream of the vicinal Au(111) substrate with a distance of ~4 cm and ~20 cm, respectively.


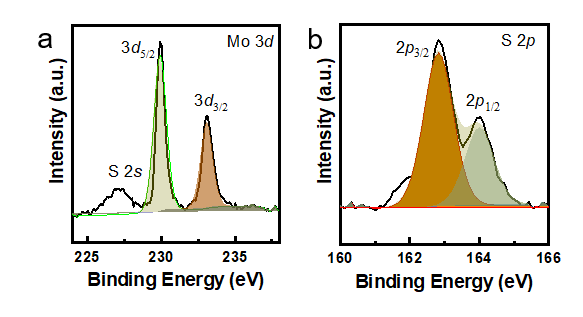


**Supplementary Fig. 2**. XPS spectra of as-grown MoS_2_ ribbons grown on Au(223) facet. (a, b) XPS spectra focused on Mo and S elements, respectively. The characteristic peaks at 229.4 eV and 232.6 eV correspond to Mo 3*d*_5/2_ and 3*d*_3/2_, and the peaks at 162.2 eV and 163.4 eV are in line with S 2*p*_3/2_ and 2*p*_1/2_, respectively, indicating the formation of MoS_2_ crystals.


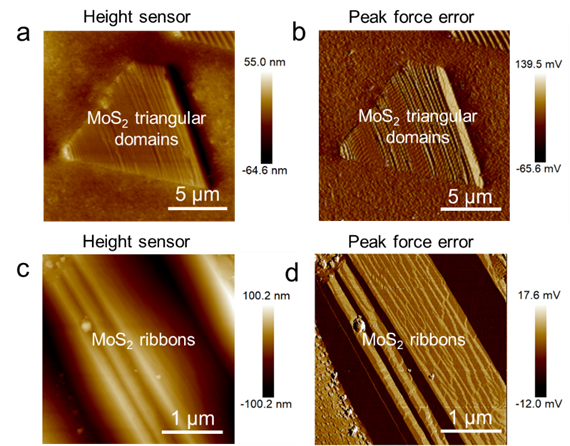


**Supplementary Fig. 3**. AFM images of as-grown monolayer MoS_2_ triangular domains and ribbons on Au substrates. (a, b) Representative height sensor and corresponding peak force error images of MoS_2_ triangular domains grown on Au substrates, respectively. (c, d) Representative height sensor and corresponding peak force error images of MoS_2_ ribbons grown on Au substrates, respectively. It can be clearly seen that, the edges of MoS_2_ flakes are always along the steps of Au substrates, providing straightforward evidence for the step-edge-guided growth mechanism.

**
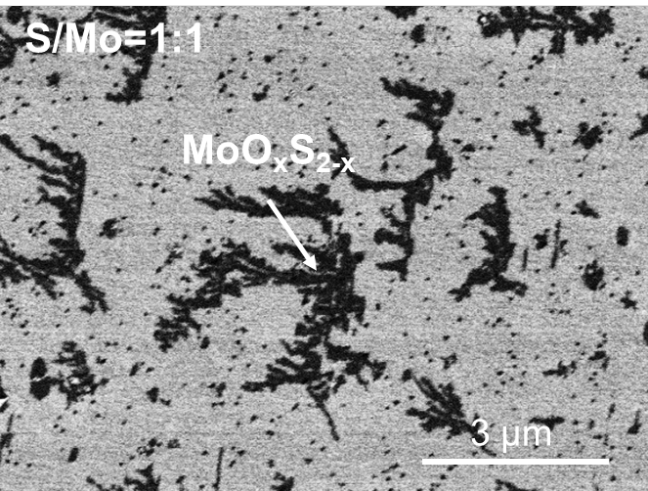
**

**Supplementary Fig. 4.** SEM images of MoO_x_S_2−x_ crystals under the S/Mo ratio of 1:1. When the S/Mo ratio is decreased to 1:1, irregularly shaped MoO_x_S_2−x_ crystals will be formed due to the insufficient supply of S precursor.

**
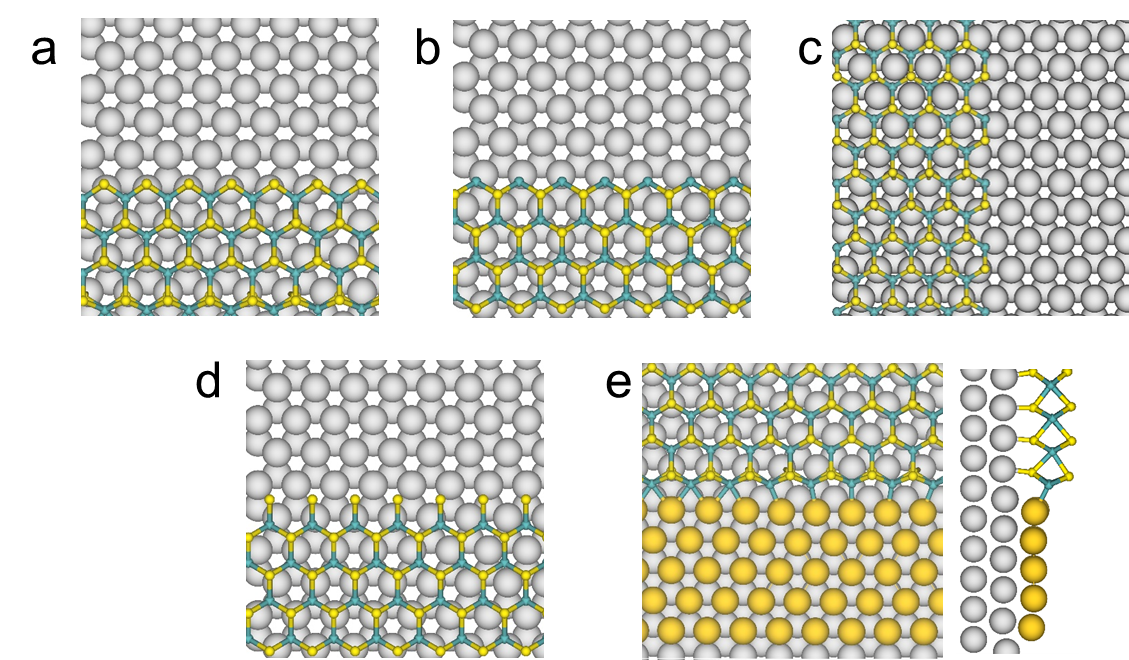
**

**Supplementary Fig. 5.** Four kinds of edge configurations of MoS_2_ ribbons. (**a‒d**) The configurations of S-zz, Mo-zz, AC, and Mo’-zz edges, respectively. **(e)** The minimal energy configuration of MoS_2_ docking to the Au <110> steps.

**
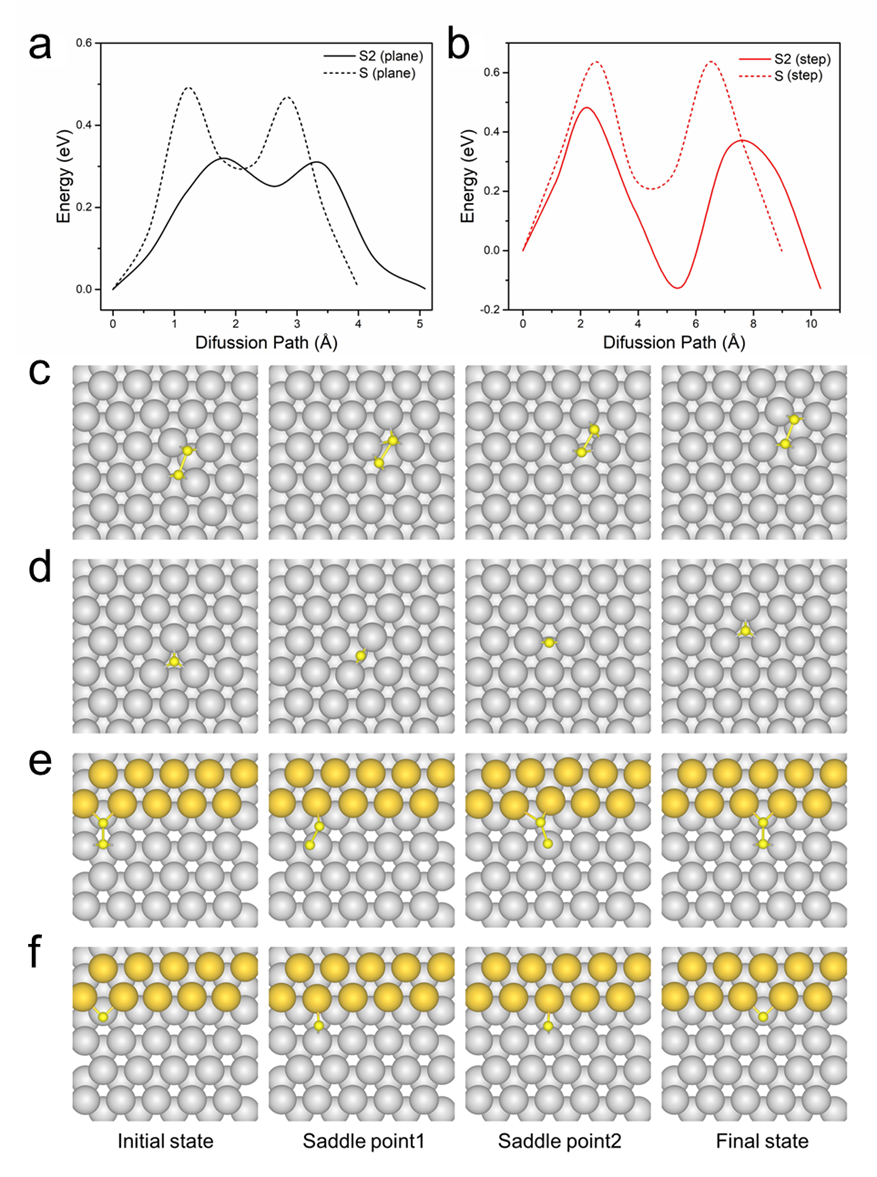
**

**Supplementary Fig. 6.** The diffusion barriers and paths of S and S2 dimmer on Au (111) plane and Au <110> steps. (**a, b**) The diffusion barriers of S and S2 dimmer on Au (111) plane and Au <110> steps, respectively. (**c, d**) The diffusion paths of S2 and S on Au (111) plane, respectively. (**e, f**) The diffusion paths of S2 and S on Au <110> steps, respectively. Atoms in grey and orange represent the Au(111) plane and stepped Au, respectively. The calculated diffusion barriers of S monomer and S2 dimer on Au (111) surface are 0.48 and 0.32 eV, indicating that S2 dimers are most likely to be the S source for MoS_2_ growth. Although the barriers of S and S2 dimmer along <110> steps are slightly higher than those on Au(111) surface, the values still remain quite low, indicating the growth of MoS_2_ was determined by the nucleation process.

**
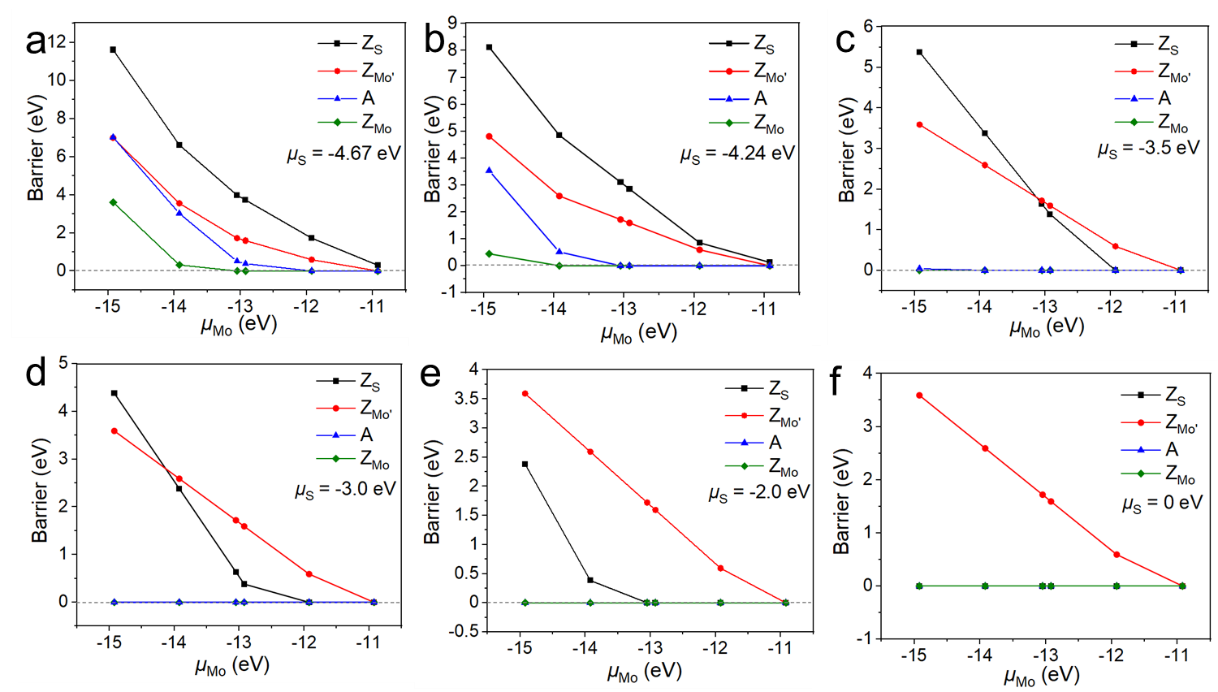
**

**Supplementary Fig. 7.** The dependence of nucleation barriers of four edges of MoS_2_ ribbons (S-zz, Mo-zz, AC, and Mo’-zz) as a function of *µ*_Mo_ at different *µ*_S_. (**a‒f**) The nucleation barriers at *µ*_S_ of ‒4.67 eV, ‒4.24 eV, ‒3.5 eV, ‒3.0 eV, ‒2.0 eV and 0 eV, respectively. As calculated, the nucleation barriers of S-zz and Mo’-zz are higher than those of AC and Mo-zz edges for all considered cases. Therefore, the kinetic model of growth can be roughly regarded as the competition between S-zz and Mo’-zz edges.


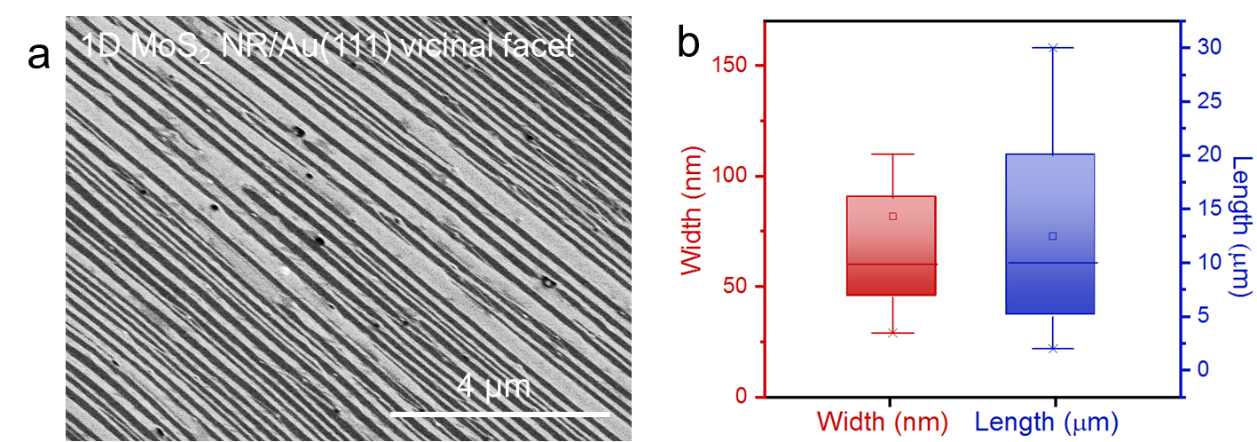


**Supplementary Fig. 8**. Large-area aligned MoS_2_ ribbon arrays grown on Au(223) facet. (a) Large-area SEM image of uniformly aligned MoS_2_ ribbons on Au(223) facet. (b) Plot of the statistical data about the widths and lengths of MoS_2_ ribbons. The widths and lengths of MoS_2_ ribbons are measured to be 20‒120 nm and 3‒30 um, respectively.


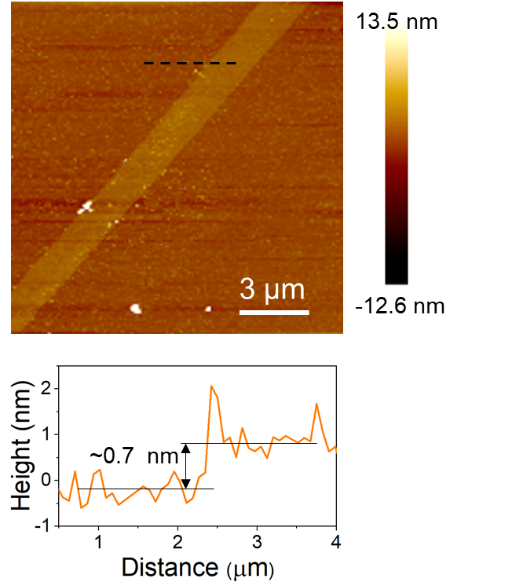


**Supplementary Fig. 9**. AFM image of a MoS_2_ ribbon transferred on SiO_2_/Si substrate. The corresponding height profile shows the thickness of MoS_2_ is ~0.7 nm, indicating its monolayer nature.


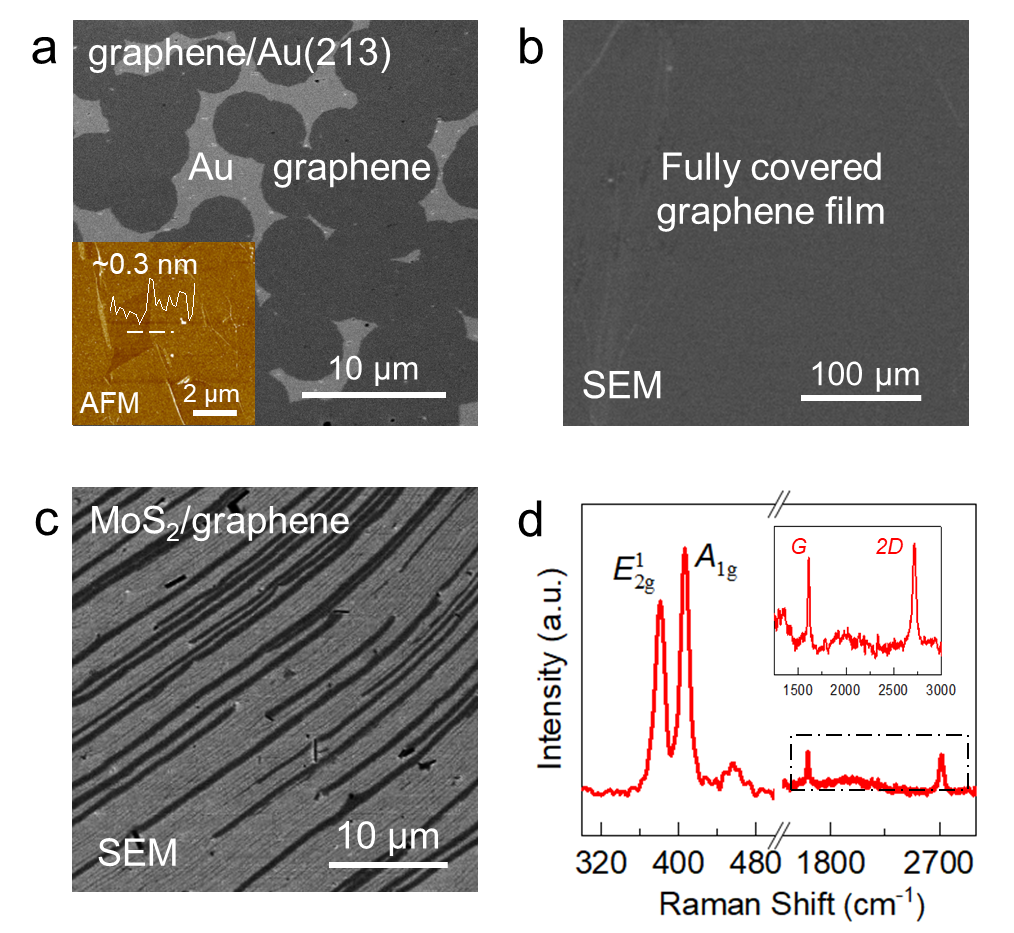


**Supplementary Fig. 10**. Monolayer graphene films and MoS_2_/graphene vertical stacks grown on Au(213) facet. (**a, b**) SEM images of the disconnected and continuous monolayer graphene film grown on Au(213) surface, respectively. Inset in a: AFM image of disconnected graphene film transferred on SiO_2_/Si substrate. The corresponding height profile shows the thickness of graphene is ~0.3 nm, indicating its monolayer nature. (**c**) SEM image of as-grown MoS_2_ ribbons on graphene covered Au(213) facet. (**d**). Representative Raman spectrum of monolayer MoS_2_ ribbons grown on monolayer graphene. The coexistence of characteristic Raman signals for monolayer MoS_2_ (*E*~386 cm^−1^ and *A*_1g_~406 cm^−1^) peaks, and graphene (*G*~1610 cm^−1^ and *2D*~2722 cm^−1^) confirms the formation of MoS_2_/graphene heterostructures.


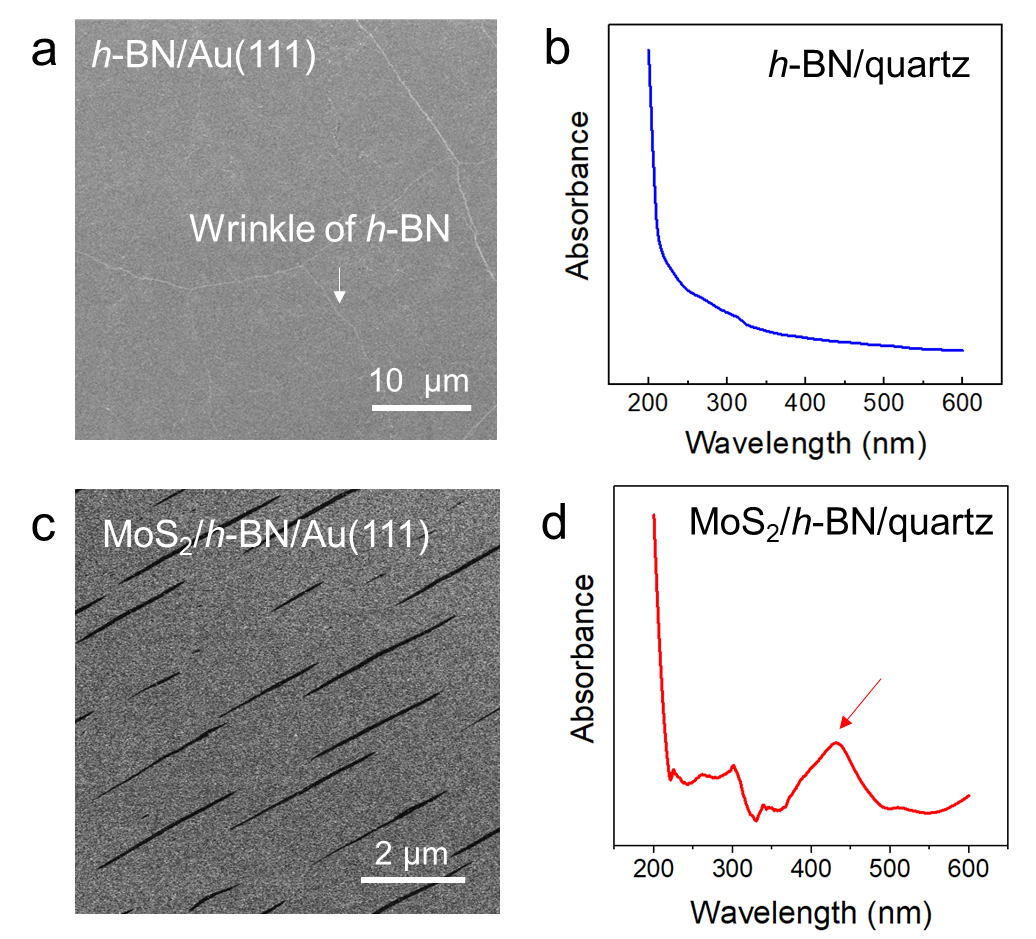


**Supplementary Fig. 11**. MoS_2_ ribbon arrays grown on monolayer *h*-BN coated-Au(111). (**a**) SEM image of monolayer *h*-BN film grown on Au(111) substrate. The uniform color contrast and formation of wrinkle (indicated by the white arrow) indicate the evolution of full-coverage *h*-BN layers. (**b**) Ultraviolet-visible (UV) absorption spectrum of monolayer *h*-BN transferred on quartz, which shows a absorption edge located at ~200 nm, confirming the formation of *h*-BN. (**c**) SEM image of MoS_2_ ribbons grown on monolayer *h*-BN covered Au(111) facet. (**d**) UV absorption spectrum of MoS_2_/*h*-BN stacks. The excitonic peak at ~430 nm (indicated by the red arrow) indicate the formation of MoS_2_ crystals on monolayer *h*-BN.


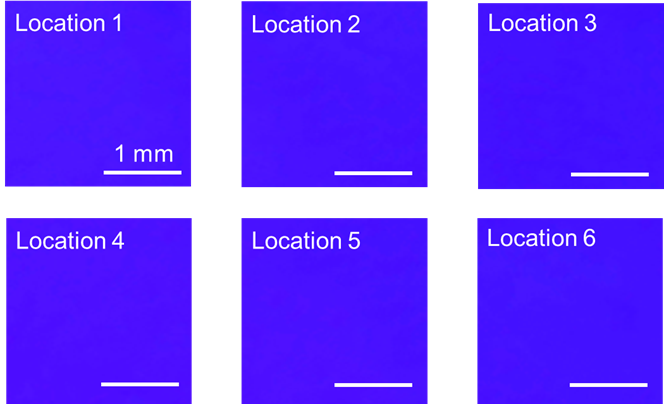


**Supplementary Fig. 12**. EBSD maps in the normal direction (ND) of the as-prepared Au(223)/W substrate, collected at six randomly selected positions. They all show homogeneous blue color contrast, confirming the single crystalline nature of the Au(223) film.


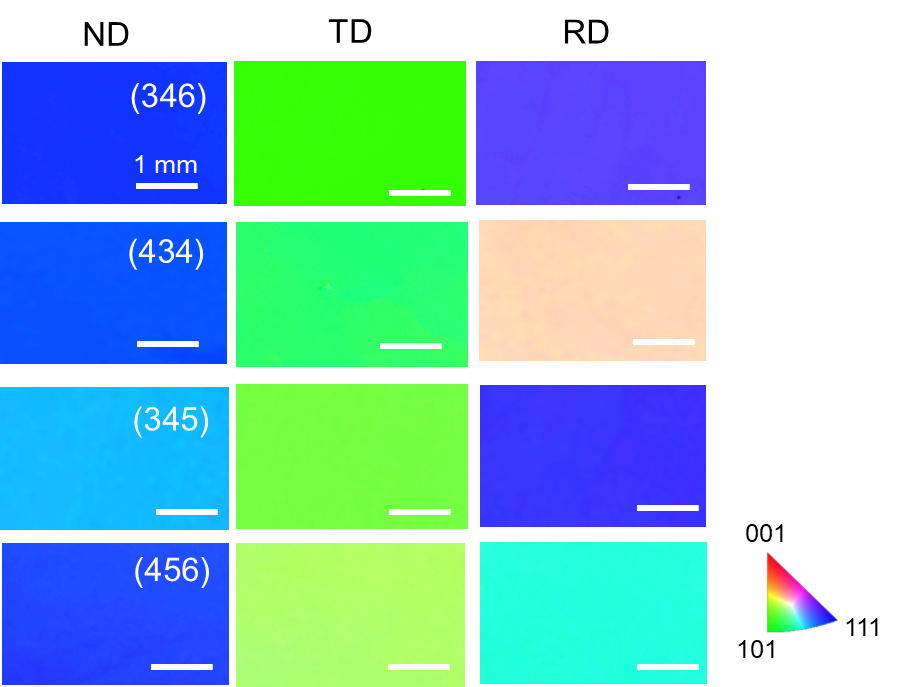


**Supplementary Fig. 13.** Representative EBSD IPF maps of as-prepared vicinal Au(111) facets. The EBSD maps in the ND, transverse direction (TD), and rolling direction (RD) all present homogenous color contrast, suggesting the single-crystalline nature of vicinal Au(111) facets.


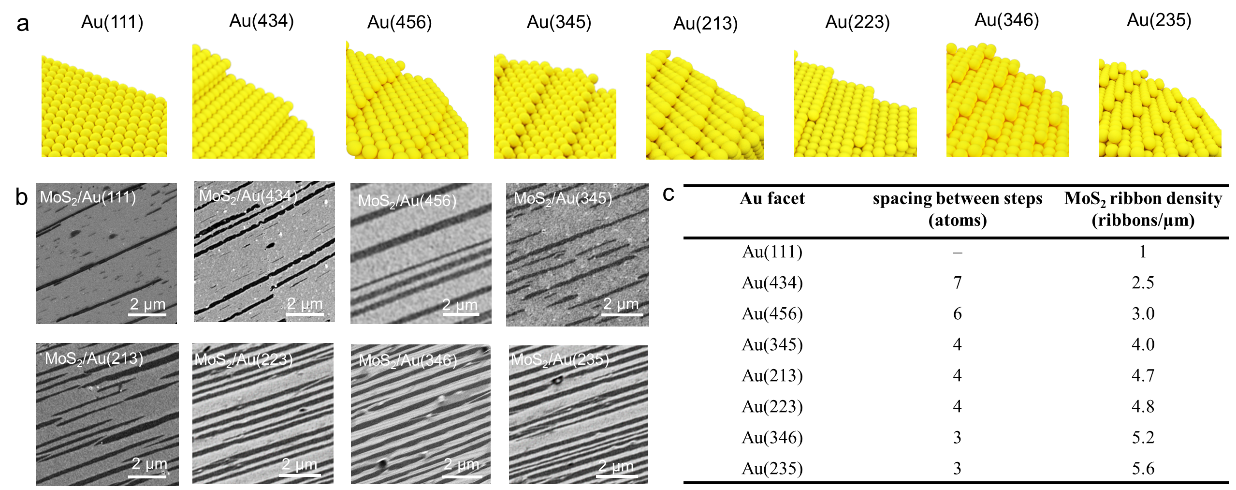


**Supplementary Fig. 14.** Morphologies of monolayer MoS_2_ ribbons grown on different high-miller-index Au facets. (**a**) Schematic illustration of representative high-miller-index Au facets vicinal to (111) with different densities of steps. (**b**) SEM images of as-grown monolayer MoS_2_ ribbons grown on high-miller-index Au facets vicinal to (111). (**c**) Comparison of the densities of MoS_2_ ribbons on various high-miller-index Au facets vicinal to (111) with different densities of steps. The as-grown monolayer MoS_2_ ribbon presents a relatively high density on the high-miller-index Au facet with a high density of step (*e.g*., Au(346), Au (235)).


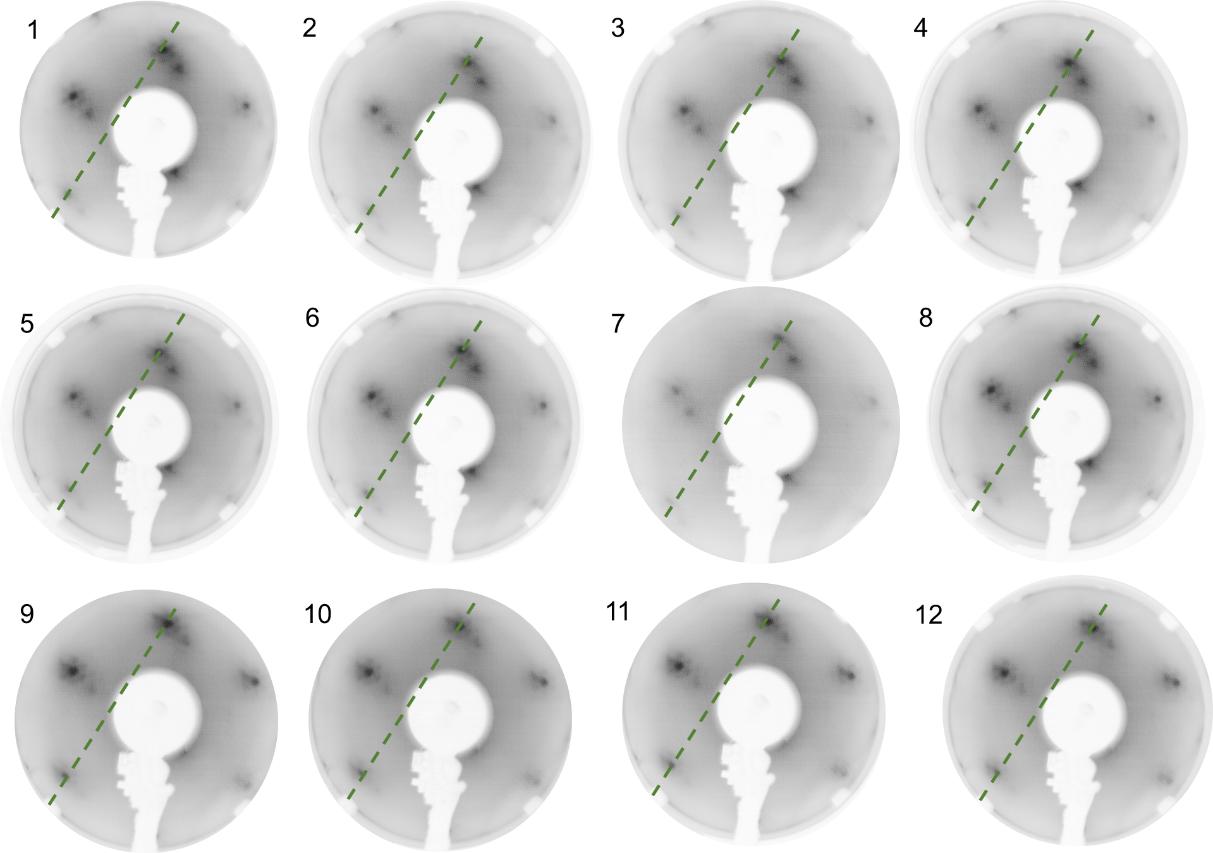


**Supplementary Fig. 15**. Representative LEED patterns from twelve randomly selected regions of the single-crystal MoS_2_ film grown on Au(223). All the patterns show an identical lattice orientation, confirming the single crystallinity of the MoS_2_ monolayer.


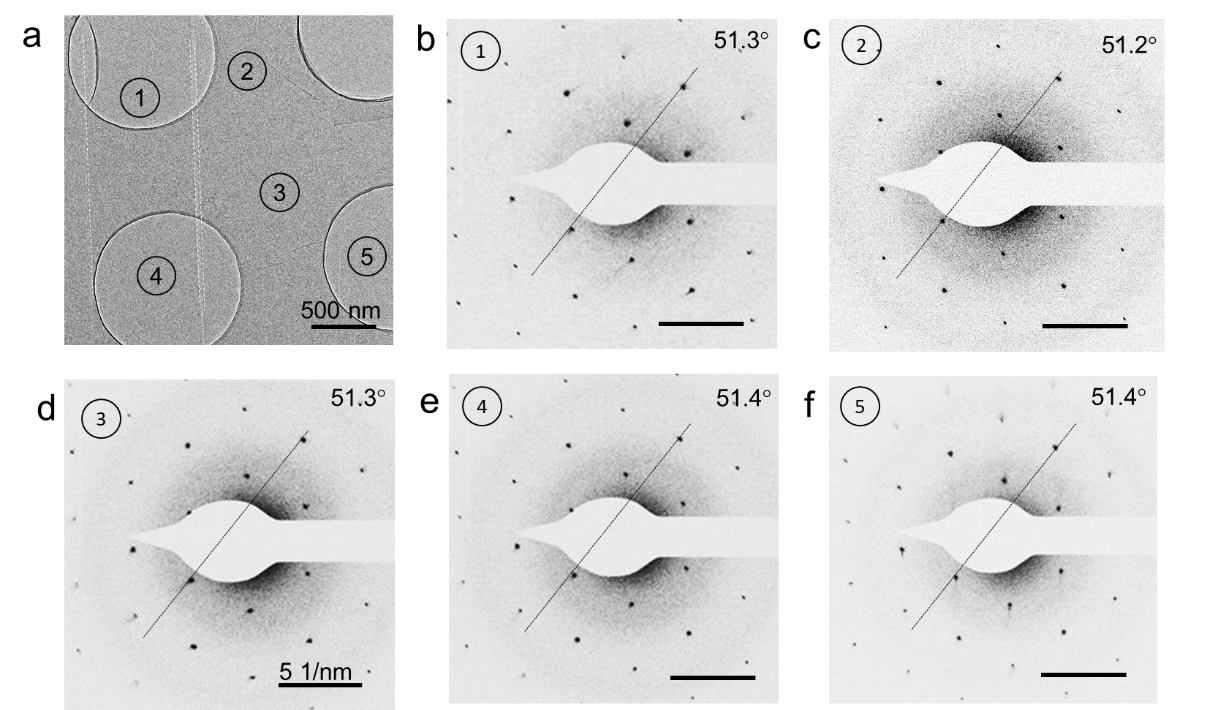


**Supplementary Fig. 16**. TEM characterizations of a nearly continuous MoS_2_ film. (**a**) Low magnified TEM image of a nearly covered MoS_2_ film on TEM grid. (**b‒f)** SAED patterns from the regions numbered 1–5 in a. The dashed lines indicate the rotation angles (51.3°, 51.2°, 51.3°, 51.4° and 51.4°) with respect to the horizontal line. The nearly identical lattice orientation (deviation smaller than ± 0.1°) indicative the single-crystal nature of the MoS_2_ film.


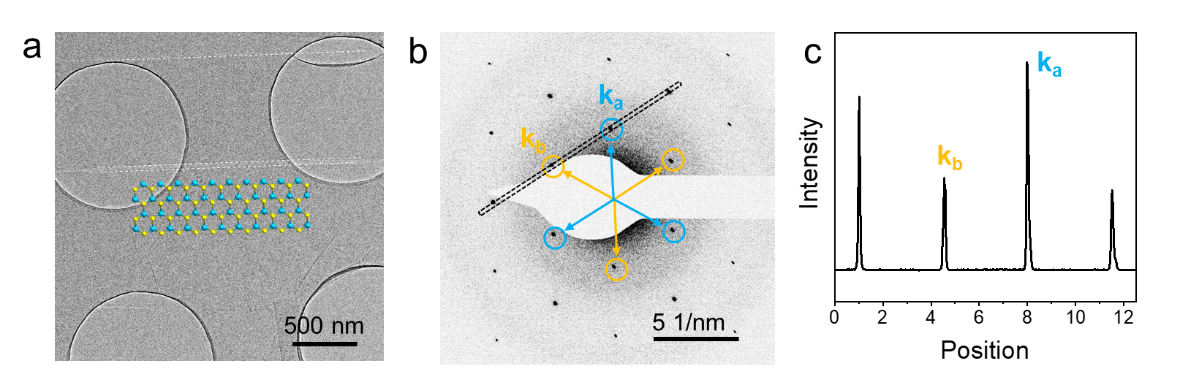


**Supplementary Fig. 17.** Characterization of the crystal structure of monolayer MoS_2_ ribbon by electron diffraction. (**a**) Low magnified TEM image of a MoS_2_ ribbon on the TEM grid. (**b**) Corresponding SAED pattern from (a), with the blue and yellow arrows marking the ***K*_a_** and ***K*_b_** spots, respectively. (**c**) Intensity line profile through the four diffraction spots circled in the dashed rectangle in panel (b).


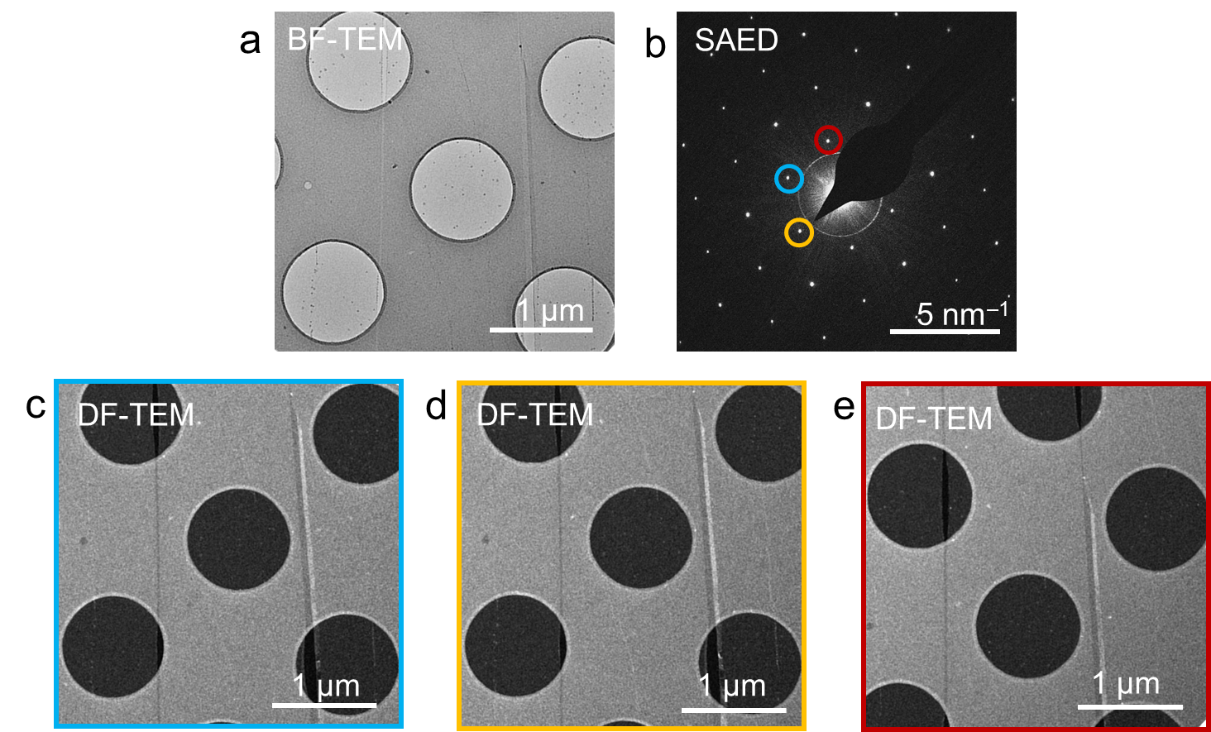


**Supplementary Fig. 18.** DF-TEM characterizations of a coalesced monolayer MoS_2_ film. (**a**) Bright-field (BF) TEM image of the MoS_2_ film. (**b**) Corresponding SAED pattern from (a). (**c‒e**) Dark-field (DF) TEM images corresponding to the red (c), blue (d) and yellow (e) spots in the diffraction image (b). The DF-TEM images show uniform intensity over the entire probed area, demonstrating the single crystallinity of the monolayer MoS_2_ film.

**
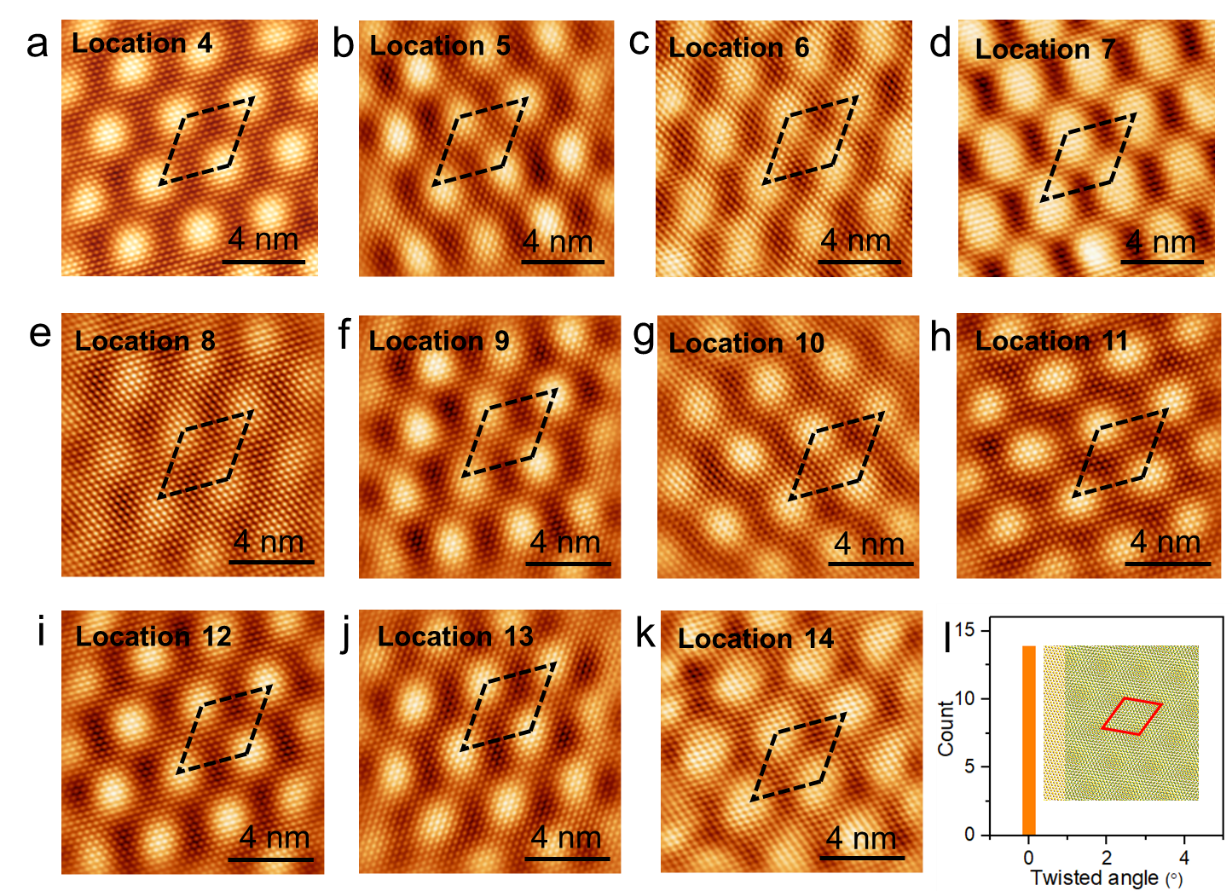
 Supplementary Fig. 19**. STM images from several randomly selected locations on the as-grown single-crystal MoS_2_ film. (**a‒k**) Representative atom-resolved STM images of the moiré patterns for MoS_2_/Au(111), presenting a fixed period of ∼3.21 ± 0.10 nm (marked by rhombus, *V*_Tip_ = −0.01 V, *I*_Tip_ = 32.79 nA; −0.003 V, 28.87 nA; −1.00 V, 4.29 nA; −0.05 V, 32.78 nA; −0.02 V, 26.25 nA; −0.02 V, 24.63 nA; −0.05 V, 34.94 nA; −0.01 V, 23.86 nA; −0.01 V, 31.76 nA; −0.01 V, 25.43 nA; −0.28 V, 7.14 nA) (**h**) Statistical distribution of the twisted angle between the MoS_2_ lattice and the moiré pattern, highly indicative the epitaxial growth behavior. Inset: simulation of the moiré pattern arising from the lattice mismatch between MoS_2_ and Au(111) terrace. STM images collected from more than 14 positions randomly selected on the monolayer film all show a nearly identical moiré period and the same lattice orientation, lacking any domain boundaries or dislocations on the entire probed area, strongly indicating the evolution of single-crystal monolayer MoS_2_ film.

**
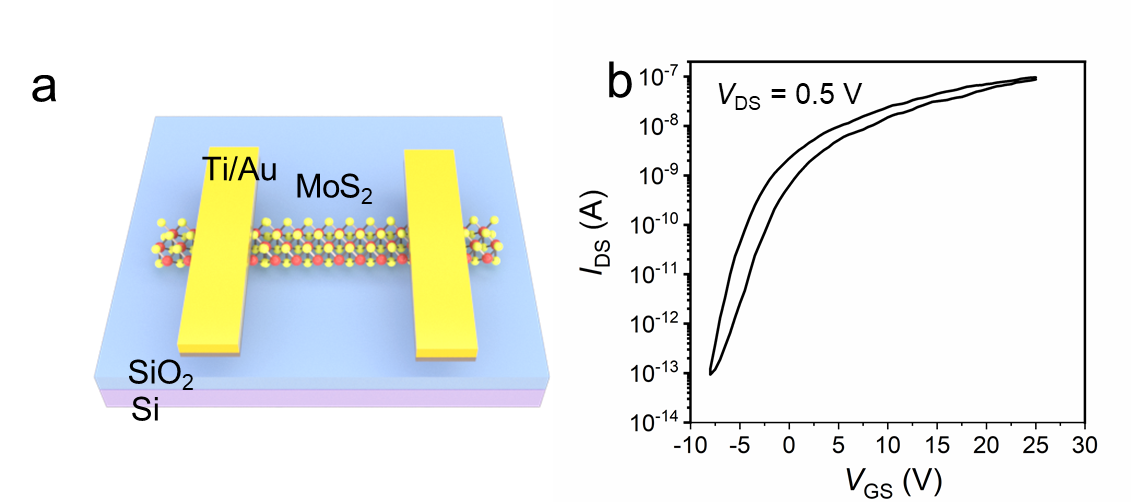
**

**Supplementary Fig. 20**. Electrical performance of a representative monolayer MoS_2_ ribbon-based FET. (a) Schematic illustration of a MoS_2_ ribbon-based device. (b) I_DS_-V_GS_ curves for a typical MoS_2_ ribbon device with the channel length/width (L/W) ratio of 700 nm/110 nm. The calculated mobility and on-off ratio are 11.8 cm^2^ V^‒1^ s^‒1^ and 10^6^, respectively.


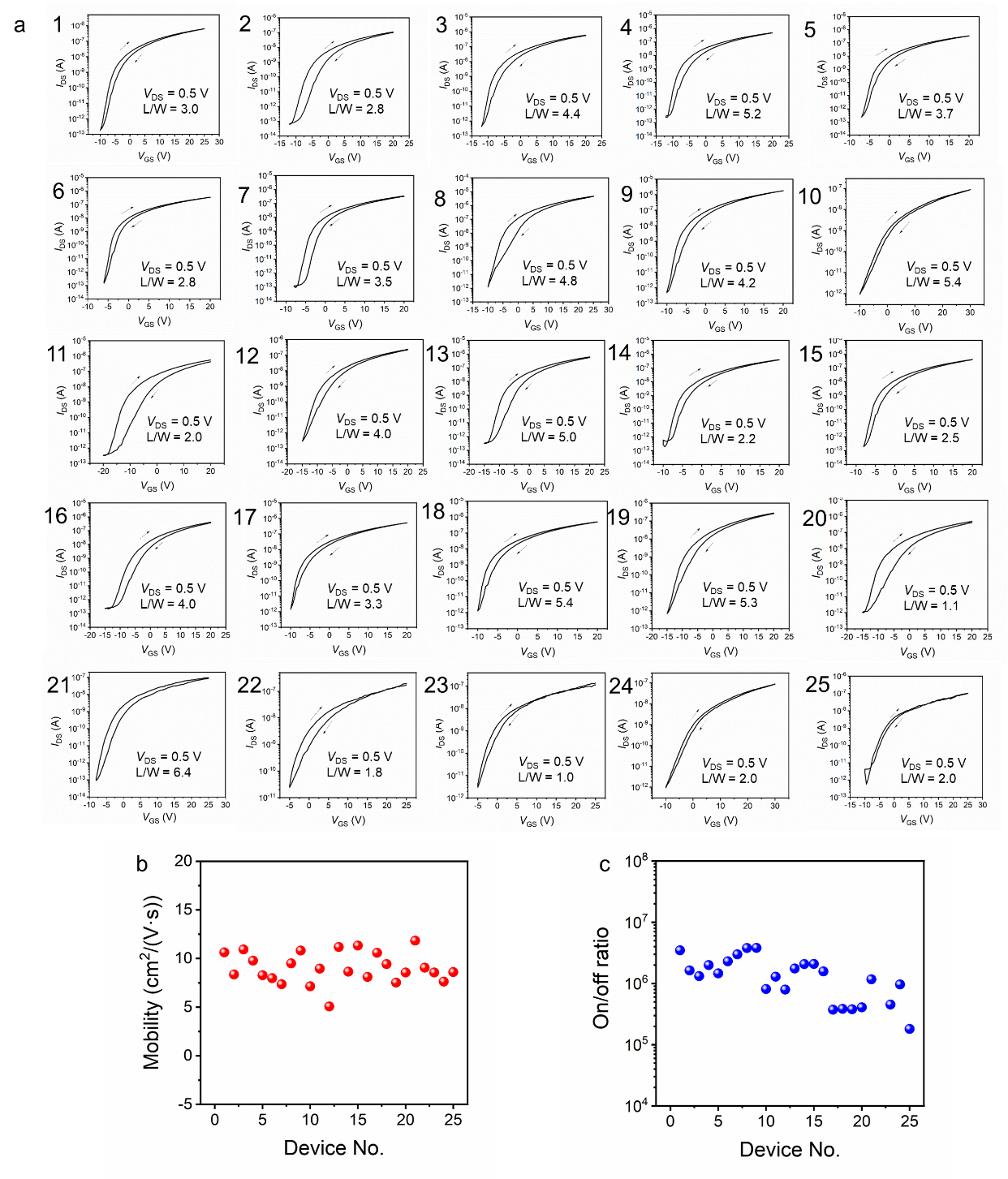


**Supplementary Fig. 21**. Statistical analysis of the electrical performances for monolayer MoS_2_ ribbon-based FETs devices. (a) All the measured transfer curves for monolayer MoS_2_ ribbon-based FET devices. (**c, d**) A summary of the carrier mobilities and the corresponding on/off ratios of 25 monolayer MoS_2_ ribbons-based FET devices. The mobilities and on/off ratios of these devices show narrow distributions of 7‒11 cm^2^ V^‒1^ s^‒1^ and 10^5^‒10^6^, respectively, confirming the high crystal-quality of the monolayer MoS_2_ ribbons grown on Au substrate and the relatively efficient transfer processes for device fabrications.


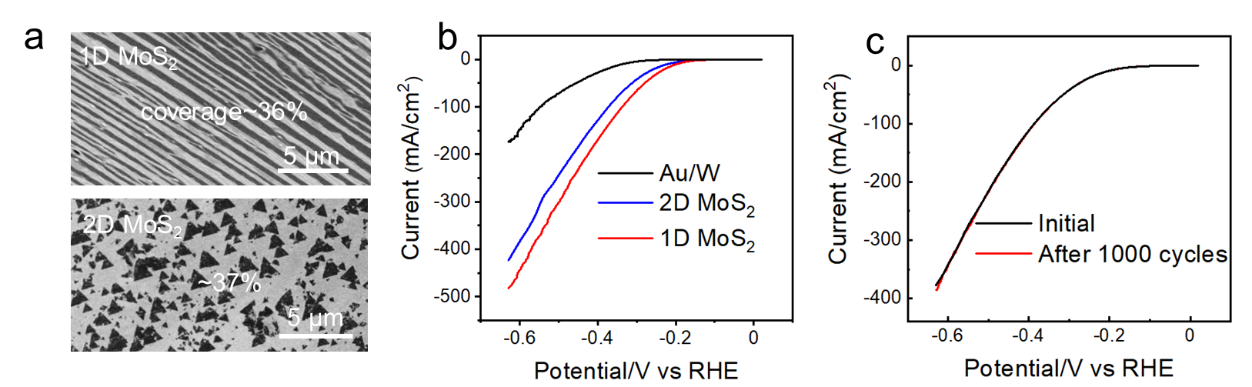


**Supplementary Fig. 22**. Electrocatalytic HER performances of as-grown MoS_2_ ribbons on Au/W. (a) SEM images of 1D MoS_2_ ribbons and 2D MoS_2_ triangles synthesized on Au/W substrates with similar coverage. (**b**) Polarization curves of 1D MoS_2_ ribbons and 2D MoS_2_ triangles. (c) Electrocatalytic durability measurements of MoS_2_ ribbons. The catalytic current density exhibits negligible loss (<5%) after 1,000 cycles, indicating the high catalytic stability.


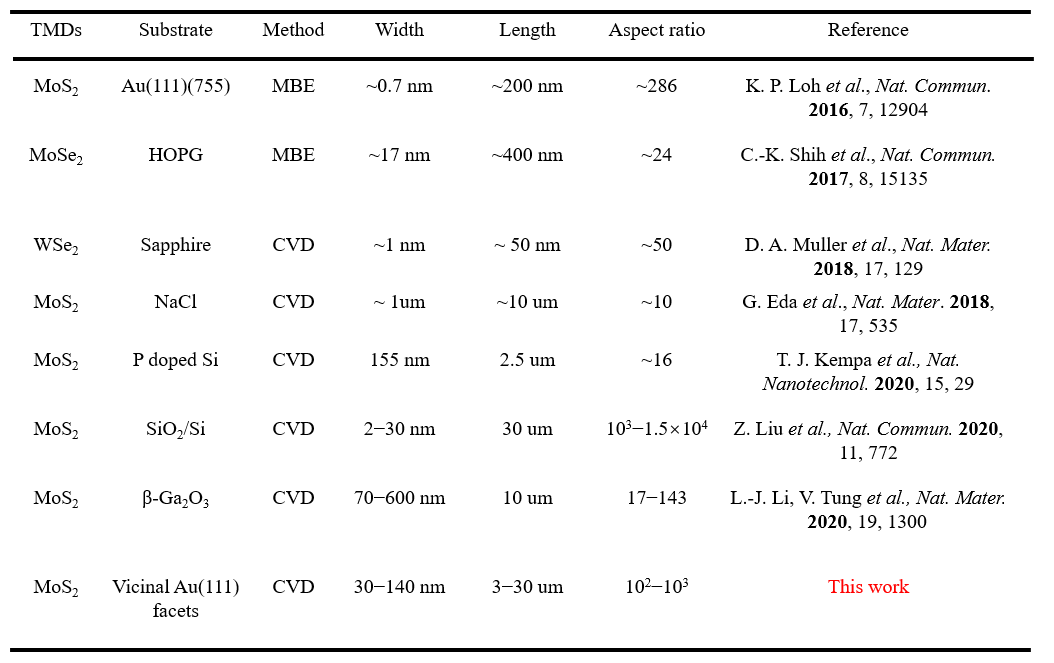


**Supplementary Table 1.** Comparison of the aspect ratios of TMDs ribbons/channels reported in the literatures with our samples.

**References**

1. J. P. Perdew, K. Burke, M. Ernzerhof, *Phys. Rev. Lett*. **77**, 3865 (1996).

2. A. Tkatchenko, M. Scheffler, *Phys. Rev. Lett*. **102**, 073005 (2009).

3. Landau, L. D.; Lifshitz, E. M. Statistical Physics, 3rd ed.; Elsevier Butterworth-Heinemann: Oxford, 1980
